# The TRAPPIII complex regulates development and virulence of *Fusarium graminearum* by coordinating autophagy and intracellular transport

**DOI:** 10.1371/journal.ppat.1013627

**Published:** 2025-10-24

**Authors:** Lei Chen, Yaxin Zhang, Geqi Zhang, Letian Xu, Mengfan Ren, Liyuan Zhang, Kai Lu, Xiaochen Chen, Yuancun Liang, Shenshen Zou, Hansong Dong

**Affiliations:** 1 Department of Plant Pathology, College of Plant Protection, Shandong Agricultural University, Tai’an, China; 2 National Key Laboratory of Wheat Improvement, Shandong Agricultural University, Tai’an, China; University of Cologne, GERMANY

## Abstract

TRAnsport Protein Particle (TRAPP) is a conserved multi-subunit tethering complex known to be involved in intracellular movement of proteins. However, its components and molecular functions in filamentous fungi remain poorly characterized. Here, we identify four TRAPPIII-specific subunits (FgTrs85, TRAPPC11, TRAPPC12, and TRAPPC13) in the phytopathogenic fungus *Fusarium graminearum*. Genetic and functional analyses reveal that FgTrs85 serves as the core subunit, collaborating with auxiliary subunits TRAPPC11, TRAPPC12, and TRAPPC13 to orchestrate fungal perithecium formation, growth, and virulence. TRAPPIII localizes to the phagophore assembly site and promotes autophagosome biogenesis by recruiting FgAtg9 through interactions involving FgTrs85 and TRAPPC13. Notably, TRAPPIII mutants exhibit more severe growth defects than autophagy-deficient strains, suggesting that the roles of TRAPPIII extend beyond autophagy. TRAPPIII regulates ER-to-Golgi and endosome-to-Golgi transport by ensuring the proper localization of secretory regulators (FgSec22, FgRud3, FgSnc1). Moreover, the overexpression of FgRab1-GTP largely suppresses all phenotypic defects associated with perithecium formation, growth, and virulence in TRAPPIII mutants, suggesting the function of TRAPPIII as a guanine nucleotide exchange factor that activates FgRab1. Altogether, our results demonstrate that TRAPPIII coordinates autophagy and intracellular transport to regulate fungal development, growth and virulence in *F. graminearum*.

## Author summary

Autophagy and intracellular transport are conserved eukaryotic pathways crucial for fungal infection of host plants. TRAPPIII is a conserved multi-subunit complex, but there are apparent differences in the composition of specific subunit across species. These variations have hindered a comprehensive understanding of TRAPPIII’s functions and have led to significant controversies regarding its roles. In this study, we identified that TRAPPIII in *Fusarium graminearum* comprises four specific subunits: FgTrs85, TRAPPC11, TRAPPC12, and TRAPPC13. FgTrs85 serves a central role, whereas TRAPPC11, TRAPPC12, and TRAPPC13 function as auxiliary components, enabling TRAPPIII to regulate autophagy and intracellular transport. TRAPPIII interacts with FgAtg9 through FgTrs85 and TRAPPC13, facilitating the anterograde transport of FgAtg9 to the PAS, thereby influencing autophagosome formation. Furthermore, TRAPPIII modulates intracellular transport by regulating the transport of FgRud3 and FgSnc1. Ultimately, we propose that the TRAPPIII-FgRab1 regulatory module plays a pivotal role in regulating fungal development, growth and virulence. Our findings elucidate the role of TRAPPIII in coordinating autophagy and intracellular transport, thereby regulating fungal development and virulence. These insights provide a novel molecular basis for the development of control strategies aimed at limiting phytopathogen infections.

## Introduction

The wheat head blight fungus *Fusarium graminearum* is a widely distributed soil-borne pathogen that infects wheat kernels, causing Fusarium head blight (FHB) and leading to significant yield losses [1,2]. In addition, this fungus produces mycotoxins that contaminate grains, posing significant immunotoxic and cytotoxic risks to both humans and animals [3,4]. Due to its devastating effects on global agriculture, economic stability, and public health, *F. graminearum* is ranked among the top ten fungal pathogens worldwide [5]. A critical phase in the FHB disease cycle is the formation and discharge of ascospores from perithecia, which serve as the primary inoculum for infecting wheat heads during flowering [6,7]. Subsequently, the ascospores germinate and rely on the apical growth of hyphae to colonize and invade the host, ultimately leading to FHB development [8,9]. Several conserved eukaryotic physiological pathways within the pathogen significantly contribute to perithecium development, hyphal growth, and host invasion [10–13]. However, the regulatory networks governing these pathways remain poorly understood in phytopathogens. Given the limited efficacy of current control strategies, elucidating the underlying molecular mechanisms in *F. graminearum* is critical for developing more effective measures targeting these physiological processes.

Autophagy, an evolutionarily conserved catabolic process essential for cellular homeostasis, plays a key role in regulating growth, development, and virulence in phytopathogenic fungi [14–16]. In *F. graminearum*, disruption of autophagy impairs virulence and perithecium formation [10]. Central to autophagy is the formation of autophagosomes (APs), which are dynamic double-membrane vesicles that sequester cellular components for degradation in the vacuole (lysosome in mammals) [17]. Atg9, the only transmembrane autophagy-related protein, mediates AP formation by facilitating the transport of Atg9 vesicles from various organelles, including the endoplasmic reticulum, mitochondria, and Golgi apparatus, to the phagophore assembly site/pre-autophagosomal structure (PAS) [18–20]. Recent studies in both yeast and mammals have identified Atg9 vesicles as seed membranes for AP formation [21–23]. Moreover, Atg9 functions as a lipid scramblase to facilitate phagophore expansion and participates in AP closure [24,25]. Despite the essential role of Atg9 in AP formation, its regulatory mechanisms exhibit species-specific variations. Notably, in *F. graminearum*, FgAtg9 requires interaction with the small GTPase FgRab7 for transport to the PAS [26], contrasting with the primary role of Rab7 in mediating AP-vacuole fusion in other eukaryotes [27]. These functional divergences underscore the necessity of elucidating autophagy mechanisms in phytopathogens.

Autophagy regulation in eukaryotes involves not only core autophagy-related proteins but also numerous components of intracellular transport pathways, revealing complex cross-regulatory mechanisms between these systems [19,28,29]. Consequently, various transport regulators influence phytopathogenic virulence by modulating both autophagy and intracellular transport. The transport protein particle (TRAPP) complex constitutes a class of multi-subunit complexes that play crucial roles in intracellular transport [30]. In yeast, four types of TRAPP complexes have been identified: TRAPPI, TRAPPII, TRAPPIII, and TRAPPIV, whereas two types, TRAPPII and TRAPPIII, have been characterized in mammals [31,32]. TRAPPIII, initially characterized in yeast, consists of the TRAPPI core complex and an additional subunit, Trs85 [33]. It functions as a guanine nucleotide exchange factor (GEF) to activate the GTPase Ypt1 (Rab1 in mammals), thereby participating in autophagy [33]. Additionally, TRAPPIII contributes to ER-to-Golgi transport [34], although its role in intracellular transport remains debated. Notably, deletion of *TRS85* in yeast does not affect growth, unlike deletions of other intracellular transport regulators, which typically cause severe defects. In mammals, TRAPPIII comprises four additional subunits compared to TRAPPI: TRAPPC8 (the Trs85 homolog), TRAPPC11, TRAPPC12, and TRAPPC13, the latter three of which are absent in yeast [32]. Despite these compositional differences, mammalian TRAPPIII also activates Rab1 and regulates autophagy. In *Magnaporthe oryzae*, the TRAPPIII-specific subunit MoTrs85 modulates autophagy and virulence [35]. However, the role of TRAPPIII in autophagy and intracellular transport in plant-pathogenic filamentous fungi remains poorly understood.

In this study, we identified the TRAPPIII complex in *F. graminearum* as comprising four specific subunits: FgTrs85, TRAPPC11, TRAPPC12, and TRAPPC13. The TRAPPIII complex regulates fungal growth, perithecium formation, and virulence. Mechanistic analyses revealed that TRAPPIII specifically localizes to the PAS, while showing no localization to APs. The interaction between TRAPPIII and FgAtg9, mediated by FgTrs85 and TRAPPC13, facilitates the anterograde transport of FgAtg9 to the PAS, thereby promoting AP biogenesis and modulating the autophagy pathway. Importantly, we found that loss-of-function mutations in TRAPPIII lead to more severe growth defects than those observed in autophagy-deficient mutants, indicating that TRAPPIII also performs autophagy-independent roles in regulating fungal growth. Furthermore, TRAPPIII disruption alters the subcellular localization of FgSec22, FgRud3, and FgSnc1, key regulators of intracellular transport that are critical for hyphal growth and virulence. In summary, this study provides a comprehensive functional characterization of TRAPPIII-specific subunits in a phytopathogenic fungus, revealing the dual role of TRAPPIII in coordinating autophagy and intracellular transport to regulate fungal development and virulence.

## Results

### Characterization of TRAPPIII-specific subunits in *F. graminearum*

**Fig 1:**
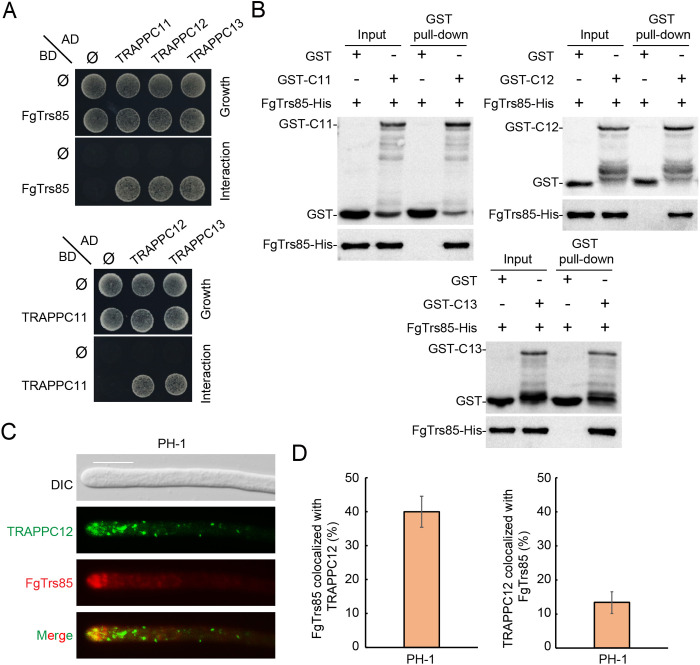
FgTrs85 interacts with three other specific subunits of the TRAPPIII complex in *F. graminearum.* **(A)** Interactions of FgTrs85 with TRAPPC11, TRAPPC12, and TRAPPC13, and of TRAPPC11 with TRAPPC12, TRAPPC13, were detected in Y2H assay. Empty plasmids (pGADT7 or pGBKT7) indicated by Ø. Yeast cells were grown on agar plates of SD-Leu-Trp (growth) and SD-Leu-Trp-His-Ade (interaction). **(B)** FgTrs85 interacts with TRAPPC11, TRAPPC12, and TRAPPC13 in GST pull-down assays. GST-tagged TRAPPC11, TRAPPC12, and TRAPPC13, along with His-tagged FgTrs85, were expressed in *E. coli*. Western blot was performed using antibodies against GST and His. **(C)** TRAPPC12-GFP colocalizes with FgTrs85-tdTomato in *F. graminearum.* PH-1 strains expressing both TRAPPC12-GFP and FgTrs85-tdTomato were grown in CM before hyphae were visualized by live-cell microscopy. Bar = 10 μm. **(D)** Quantification of the co-localization between TRAPPC12-GFP and FgTrs85-tdTomato from panel **C.** Left, percentage of co-localization of FgTrs85-tdTomato with TRAPPC12-GFP, and the number of red dots used for the analysis. Right, percentage of co-localization of TRAPPC12-GFP with FgTrs85-tdTomato, and the number of green dots used for the analysis. More than 50 hyphae were examined; the error bars in the graph represent the standard deviation (SD) from three independent experiments.

Trs85 is a TRAPPIII-specific subunit conserved across eukaryotes, from yeast to mammals. In *F. graminearum*, the homologous protein is encoded by the FGSG_04266 gene and is referred hereby as *FgTRS85*. To identify potential additional TRAPPIII-specific components, we conducted affinity capture followed by mass spectrometry (MS) using FgTrs85 as a bait. This analysis identified 14 potential interactors (S3 Table), including four conserved TRAPP core subunits: FGSG_02649, FGSG_06941, FGSG_05490 and FGSG_01124. These subunits are homologous to *Saccharomyces cerevisiae* Bet3, Bet5, Trs20, and Trs23, respectively. Additionally, three other proteins encoded by FGSG_08748, FGSG_04046, and FGSG_12900 were also identified. Although these proteins are absent in yeast, they exhibit homology to mammalian TRAPPIII-specific subunits TRAPPC11, TRAPPC12, and TRAPPC13, respectively (S3 Table). Phylogenetic analysis revealed that FgTrs85, TRAPPC11, TRAPPC12, and TRAPPC13 from filamentous fungi form a closely related evolutionary clade (S1 Fig). To assess whether these proteins function together as TRAPPIII-specific subunits in *F. graminearum*, we performed yeast two-hybrid (Y2H), GST pull-down and Co-immunoprecipitation (Co-IP) assays. FgTrs85 interacted with TRAPPC11, TRAPPC12, and TRAPPC13 (Figs 1A, 1B and S2). Furthermore, TRAPPC11 also showed interactions with both TRAPPC12 and TRAPPC13 (Fig 1A), supporting the presence of a protein interaction network among these TRAPPIII-specific subunits. Next, we investigated their subcellular localization. Attempts to tag TRAPPC11 and TRAPPC13 with fluorescent proteins at either the N- or C-terminus failed to complement the growth defects of their respective deletion mutants, suggesting that these fusions were non-functional. However, TRAPPC12-GFP retained functionality and was used for co-localization studies with FgTrs85-tdTomato. Approximately 40% of FgTrs85-tdTomato puncta co-localized with TRAPPC12-GFP, whereas about 13% of TRAPPC12-GFP signals co-localized with FgTrs85-tdTomato (Fig 1C and 1D), indicating a partial but specific co-localization. Taken together, these results support that *F. graminearum* has TRAPPIII-specific subunits comprising FgTrs85, TRAPPC11, TRAPPC12, and TRAPPC13, resembling the composition of the mammalian TRAPPIII complex and distinct from that of yeast.

### The TRAPPIII complex is required for the growth, development and virulence of *F. graminearum*

**Fig 2:**
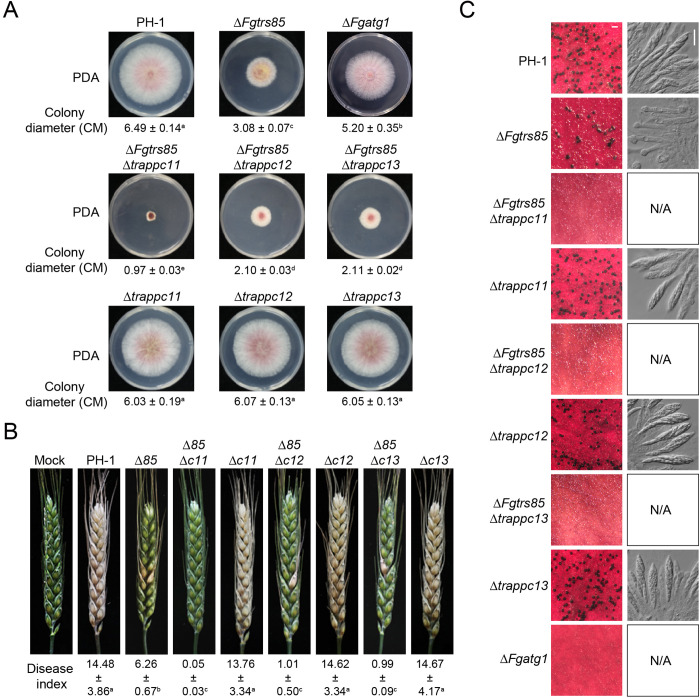
The TRAPPIII complex is critical for vegetative growth, perithecium formation and full virulence of *F. graminearum.* **(A)** TRAPPIII-specific subunit mutants exhibit vegetative growth defects. PH-1 and mutant strains were cultured on PDA for 3 days; colony diameters are shown below the images. **(B)** TRAPPIII-specific subunit mutants exhibit defects in perithecium formation during sexual reproduction. PH-1 and mutant strains were cultured on carrot agar plates to detect perithecia production; microscope observation of ascospores extruded from perithecia. Bar = 500 μm (left) and 20 μm (right). **(C)** TRAPPIII-specific subunit mutants exhibit a severe loss of virulence during plant infection. Conidial suspensions of PH-1 and mutant strains were inoculated into flowering wheat heads; the disease index for each strain is shown below the images. Results represent three independent experiments. ± represent SD. The same letters indicate no statistical significance, while different letters indicate significant difference (p < 0.01).

To elucidate the functional roles of TRAPPIII and its specific subunits in *F. graminearum*, we generated a series of deletion mutants targeting the TRAPPIII-specific subunits (S3, S4A and S4B Figs). Deletion of *FgTRS85* significantly impaired growth, whereas the deletion of TRAPPC11, TRAPPC12, or TRAPPC13 individually did not affect growth, with these mutants displaying phenotypes similar to the wild-type PH-1 and Δ*Fgtrs85/FgTRS85* strain (Figs 2A and S5). Notably, double mutants combining *FgTRS85* deletion with the deletion of any of the three TRAPPIII-specific subunits exhibited more severe growth defects than the Δ*Fgtrs85* single mutant (Fig 2A), and the growth defects of the double mutants were rescued by reintroduction of the corresponding genes (S5 Fig). Interestingly, the Δ*trappc11*Δ*trappc12*Δ*trappc13* triple mutant retained normal growth (Figs 2A and S4C). These observations suggest that while FgTrs85 plays a central role in TRAPPIII function during growth, TRAPPC11, TRAPPC12, and TRAPPC13 may function in a supporting or redundant manner.

In virulence assays, the Δ*trappc11*, Δ*trappc12*, and Δ*trappc13* mutants retained full virulence, consistent with the PH-1 strain. In contrast, the Δ*Fgtrs85* mutant exhibited markedly reduced virulence, with a disease index of 6.26 (Fig 2B). Furthermore, the double mutant strains (Δ*Fgtrs85*Δ*trappc11*, Δ*Fgtrs85*Δ*trappc12*, and Δ*Fgtrs85*Δ*trappc13*) showed further attenuation in virulence relative to the Δ*Fgtrs85* mutant (Fig 2B), while the Δ*trappc11*Δ*trappc12*Δ*trappc13* triple mutant remained fully virulent (S4D Fig). Since DON is a significant virulence factor synthesized in toxisomes, we quantified its production in these strains. All mutants produced less DON compared to PH-1, with double mutants displaying more pronounced reductions than single mutants (S6A Fig). Consistent with this, toxisome formation monitored via Tri4-GFP localization was more disrupted in the double mutants than in the single mutants (S6B Fig). These data suggest that TRAPPIII is essential for DON biosynthesis and virulence, with specific subunits contributing in a manner consistent with their roles during vegetative growth.

We next assessed the role of TRAPPIII in sexual reproduction, given that ascospores are the primary inoculum for FHB outbreaks. The Δ*trappc11*, Δ*trappc12* and Δ*trappc13* mutants produced normal perithecia and ascospores, while the Δ*Fgtrs85* mutants formed perithecia but generated malformed ascospores (Fig 2C). Importantly, the double mutants Δ*Fgtrs85*Δ*trappc11*, Δ*Fgtrs85*Δ*trappc12* and Δ*Fgtrs85*Δ*trappc13* failed to produce perithecia, indicating a more severe defect in sexual development (Fig 2C). Asexual reproduction was also compromised in these mutants, with double mutants showing significantly reduced conidial production, shorter conidia, and impaired germination compared to single mutants (S7A–S7D Fig). These results demonstrate that TRAPPIII is involved in regulating both sexual and asexual reproduction in *F. graminearum*.

### TRAPPIII is essential for autophagy in *F. graminearum*

**Fig 3:**
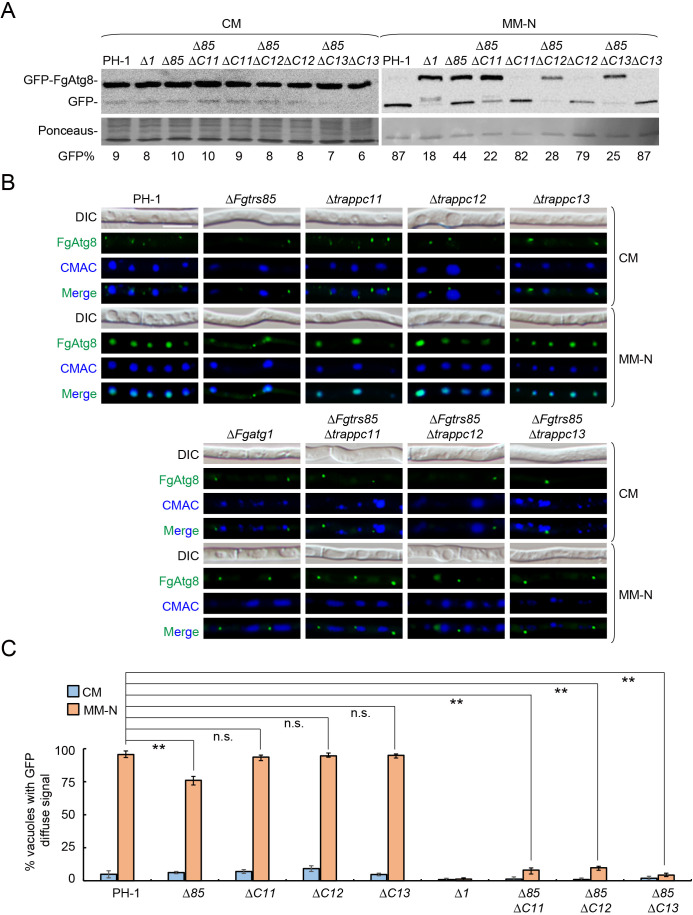
The TRAPPIII complex is required for autophagy of *F. graminearum.* **(A)** TRAPPIII-specific subunit mutants exhibit a defect in GFP-FgAtg8 processing under starvation. WT and mutant vegetative hyphae expressing GFP-FgAtg8 were grown in CM medium and shifted to nitrogen starvation medium (MM-N). Protein extracts from vegetative hyphae lysates were resolved by immunoblot analysis using anti-GFP antibodies; the percentage of free GFP in each lane is shown under the blot. **(B)** TRAPPIII-specific subunit mutants exhibit a defect transport of GFP-FgAtg8 to the vacuole. WT and mutant vegetative hyphae expressing GFP-FgAtg8 were examined by fluorescence microscopy under CM or MM-N medium. CMAC was used to stain the vacuole before vegetative hyphae were visualized by microscopy. Bar = 10 μm. **(C)** Results from panel B were quantified from more than 200 cells. The percentage of cells with GFP-FgAtg8 transport to the vacuole is shown. Error bars represent SD. Results represent three independent experiments. **P < 0.01; N.S., no significance.

Autophagy is known to be critical for perithecium development and virulence in *F. graminearum* [10]. Although the role of TRAPPIII in this process is highly conserved [33], how its specific subunits function in *F. graminearum* remains unclear. Here, we assessed TRAPPIII-mediated autophagy by quantifying autophagic flux using a GFP–FgAtg8 processing assay. Under non-starvation conditions, GFP-FgAtg8 remained intact in all strains (Fig 3A). Upon starvation, free GFP was detected in the PH-1, Δ*trappc11*, Δ*trappc12*, and Δ*trappc13* strains, indicating normal autophagy. In contrast, the Δ*Fgtrs85* and all double mutants retained the GFP-FgAtg8 fusion, similar to the Δ*Fgatg1* strain, indicating impaired autophagic flux (Fig 3A). Notably, autophagy defects were more pronounced in the double mutants than in the Δ*Fgtrs85* single mutant. Live-cell fluorescence imaging of GFP-FgAtg8 revealed punctate cytoplasmic localization under nutrient-rich conditions in all strains, without vacuolar co-localization (Fig 3B and 3C). Following starvation, GFP-FgAtg8 was efficiently transported to the vacuole in PH-1, Δ*trappc11*, Δ*trappc12*, and Δ*trappc13*, indicating functional autophagy. However, in the Δ*Fgtrs85* strain, GFP-FgAtg8 partially accumulated in the cytoplasm, with only some reaching the vacuole. In contrast, the double mutants exhibited complete failure of vacuolar delivery, with GFP-FgAtg8 retained entirely as cytoplasmic puncta (Fig 3B and 3C). These results confirm that FgTrs85 is required for autophagy, and that the additional loss of TRAPPC11, TRAPPC12, or TRAPPC13 fully blocks autophagy flux, revealing cooperative functions among the TRAPPIII-specific subunits in this essential pathway.

### TRAPPIII localizes to the PAS

**Fig 4:**
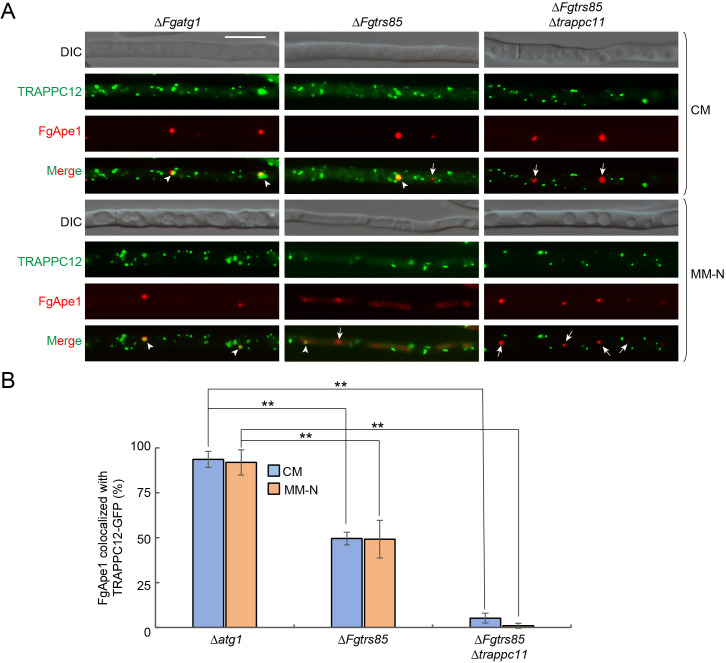
TRAPPIII complex localizes to the PAS of *F. graminearum.* **(A)** TRAPPC12 localization to the PAS is dependent on both FgTra85 and TRAPPC11. The Δ*Fgatg1*, Δ*Fgtrs85*, and Δ*Fgtrs85*Δ*TRAPPC11* strains expressing both TRAPPC12-GFP and RFP-FgApe1 were examined by fluorescence microscopy under CM or MM-N conditions. Arrowheads indicate FgApe1 puncta that colocalize with TRAPPC12. Arrows indicate non-colocalized RFP-FgApe1 puncta. Bar = 10 μm. **(B)** The colocalization of RFP-FgApe1 with TRAPPC12-GFP were quantified from panel A, with more than 300 RFP-FgApe1 puncta examined in each strain. Error bars represent SD. Results represent three independent experiments. **P < 0.01.

AP formation is believed to initiate at the PAS [17]. To examine whether TRAPPIII localizes to the PAS in *F. graminearum*, we tracked TRAPPC12-GFP using RFP-FgApe1 as a PAS marker. To improve the visibility of the PAS, localization was assessed in the Δ*Fgatg1* background. As shown in Fig 4, over 90% of RFP-FgApe1 puncta co-localized with TRAPPC12-GFP under both nutrient-rich and starvation conditions, indicating that TRAPPIII localizes to the PAS and may regulate autophagy from this site. In the Δ*Fgtrs85* mutant, the PAS localization of TRAPPC12-GFP was reduced to approximately 50%, and in the Δ*Fgtrs85*Δ*trappc11* double mutant, co-localization dropped below 3%. Consistent with this result, TRAPPC12-GFP also failed to localize to the PAS in the Δ*Fgtrs85*Δ*trappc13* double mutant (S8 Fig). These data indicate that while FgTrs85 is partially required for TRAPPIII localization to the PAS, the combined loss of FgTrs85 and TRAPPC11/13 nearly abolishes this localization, likely contributing to the more severe autophagy defects observed in the double mutant. We further investigated whether FgTrs85 itself also localizes to the PAS. The results showed that FgTrs85-mNeoGreen co-localized with RFP-FgApe1 in both the Δ*Fgatg1* and Δ*Fgatg1*Δ*trappc12* mutants (S9 Fig), indicating that the PAS localization of FgTrs85 is independent of TRAPPC12, consistent with normal autophagy in the Δ*trappc12* mutant. Collectively, these findings suggest that TRAPPIII functions at the PAS to regulate autophagy in *F. graminearum*.

### TRAPPIII is required for AP formation in *F. graminearum*

**Fig 5:**
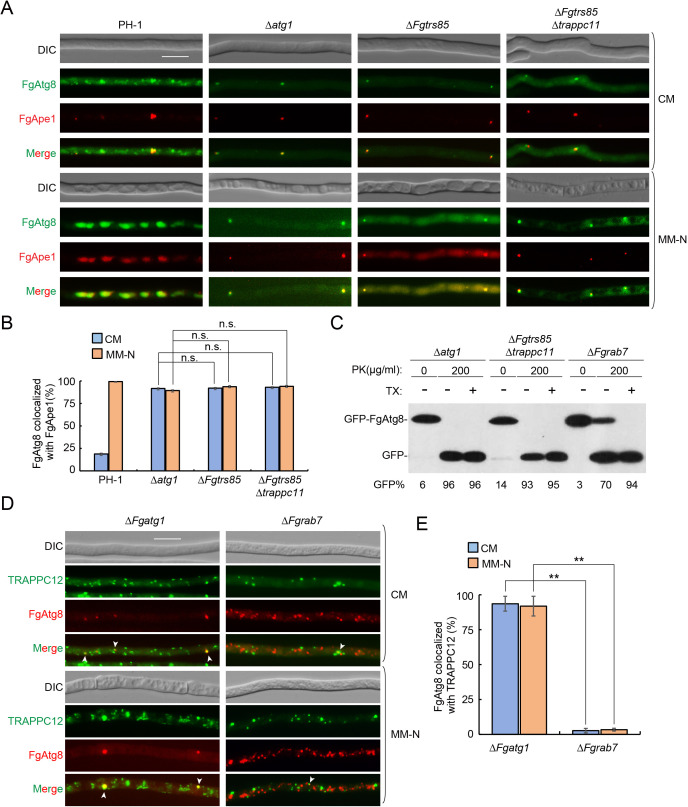
TRAPPIII complex plays an essential role in AP formation of *F. graminearum.* **(A)** TRAPPIII is not involved in the transport of FgAtg8 to PAS. The PH-1, Δ*Fgatg1*, Δ*Fgtrs85*, and Δ*Fgtrs85*Δ*TRAPPC11* strains expressing both GFP-FgAtg8 and RFP-FgApe1 were examined by fluorescence microscopy in CM or MM-N medium. Bar = 10 μm. **(B)** The colocalization of GFP-FgAtg8 with RFP-FgApe1 was quantified from panel A, and more than 300 GFP-FgAtg8 puncta in each strain were examined. Error bars represent SD. Results represent three independent experiments. N.S., no significance. **(C)** The Δ*Fgtrs85*Δ*TRAPPC11* mutant was defective in AP formation. Fractions were extracted from the hyphae of Δ*Fgatg1*, Δ*Fgtrs85*Δ*TRAPPC11* and Δ*Fgrab7* mutant strains expressing GFP-Atg8, and subjected to protease (PK)-protection assays, followed by immunoblot analysis. The fractions containing GFP-FgAtg8 were treated before (0 μg/ml) and after the addition of PK (200 μg/ml), with or without addition of detergent (Triton X-100; TX) (see Materials and methods). **(D)** TRAPPIII is localized to the PAS, but not to APs. The Δ*Fgatg1* and Δ*Fgrab7* strains expressing both TRAPPC12-GFP and mCherry-FgAtg8 were grown in CM and then shifted to MM-N (4 h) before visualizing the hyphae using live-cell microscopy. Arrowheads indicate TRAPPC12 dots that colocalize with FgAtg8. Bar = 10 μm. **(E)** Results from panel D were quantified for the co-localization of mCherry-FgAtg8 with TRAPPC12-GFP. More than 50 hyphae per strain were examined; the data are presented as the mean ± SD of each variable from three independent experiments. **P < 0.01; N.S., no significance.

To better understand the autophagy defects in TRAPPIII-specific subunit mutants, we analyzed GFP-FgAtg8 localization to determine the which steps in the autophagy pathway were affected. In strains lacking functional TRAPPIII, GFP-FgAtg8 accumulated in cytoplasmic puncta, consistent with blocked autophagy transport. To test whether TRAPPIII is involved in recruiting Atg8 to the PAS, we examined co-localization of GFP-FgAtg8 with RFP-FgApe1. In PH-1, approximately 20% of GFP-FgAtg8 puncta co-localized with RFP-FgApe1 under non-starvation conditions, and both were efficiently transported to the vacuole during nitrogen starvation (Fig 5A and 5B). In contrast, in the Δ*Fgtrs85*Δ*trappc11* mutant, over 90% of GFP-FgAtg8 puncta co-localized with RFP-FgApe1 under both conditions, indicating that the recruitment of FgAtg8 to the PAS is not disrupted (Fig 5A and 5B). However, GFP-FgAtg8 failed to be transported to the vacuole in this mutant, similar to Δ*Fgatg1*, which serves as the autophagy-deficient control. These data suggest that TRAPPIII is not required for Atg8 recruitment to the PAS.

Atg8 is a conserved autophagy marker that localizes to the PAS, is incorporated into APs, and is ultimately delivered to the vacuole. Although FgAtg8 localized normally to the PAS in the Δ*Fgtrs85*Δ*trappc11* mutant, its vacuolar transport was blocked (Fig 5A and 5B). To further evaluate AP biogenesis in this mutant, we performed a protease protection assay. Membrane fractions containing APs or PAS were isolated, and the sensitivity of GFP-FgAtg8 to exogenous proteinase K (PK) was assessed by immunoblotting. In the Δ*Fgatg1* mutant, which cannot form intact APs, GFP-FgAtg8 was fully digested by PK (Fig 5C). Conversely, in the Δ*Fgrab7* mutant, which accumulates intact APs, a portion of GFP-FgAtg8 was protected from protease digestion, indicating successful membrane encapsulation. The Δ*Fgtrs85*Δ*trappc11* mutant displayed a digestion pattern similar to that of Δ*Fgatg1* (Fig 5C), suggesting a failure in AP biogenesis. These findings support a model in which TRAPPIII is essential for AP formation in *F. graminearum*.

Many proteins involved in AP biogenesis, including Atg proteins, transiently localize to the PAS or phagophore and then dissociate from mature APs [36,37]. To investigate whether TRAPPIII exhibits a similar localization pattern, we analyzed the co-localization of TRAPPC12-GFP and mCherry-FgAtg8 in different genetic backgrounds. In the Δ*Fgatg1* mutant, which lacks the formation of APs, mCherry-FgAtg8 localized to the PAS, with over 90% co-localization with TRAPPC12-GFP observed under both nutrient-rich and starvation conditions (Fig 5D and 5E), thereby confirming the PAS localization of TRAPPIII. In contrast, in the Δ*Fgrab7* mutant, where mature APs accumulate, mCherry-FgAtg8 localized to APs, with less than 4% co-localization with TRAPPC12-GFP under both conditions (Fig 5D and 5E), indicating that TRAPPIII does not associate with mature APs.

Overall, our data indicate that TRAPPIII localizes to the PAS in *F. graminearum* to regulate AP biogenesis and maturation, subsequently dissociating from the structure prior to the completion of AP.

### TRAPPIII complex recruits FgAtg9 to the PAS in *F. graminearum*

**Fig 6:**
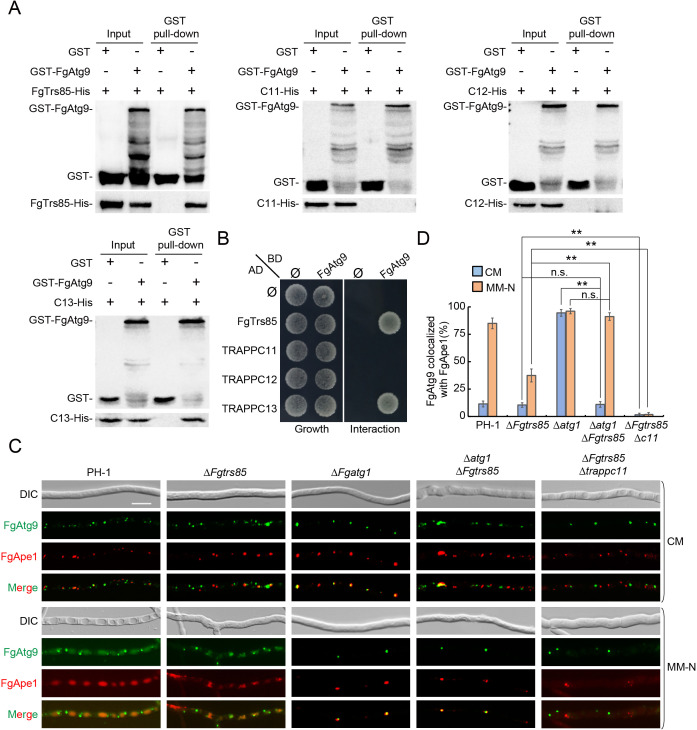
*F. graminearum* TRAPPIII complex recruits Atg9 to the PAS. **(A)** FgTrs85 and TRAPPC13, but not TRAPPC11 and TRAPPC12, interact with FgAtg9. In vitro GST pull-down assays were performed using GST-tagged FgAtg9 and His-tagged FgTrs85, TRAPPC11/12/13 were expressed in *E. coli*. Western blot was performed using antibodies against GST and His. **(B)** FgTrs85 and TRAPPC13, but not TRAPPC11 or TRAPPC12, can interact with FgAtg9 in Y2H assay. Empty plasmids (pGADT7 or pGBKT7) are indicated by Ø. Yeast cells were grown on agar plates of SD-Leu-Trp (growth) or SD-Leu-Trp-His-Ade (interaction). **(C)** TRAPPIII is required for the anterograde transport of FgAtg9 to PAS. PH-1, Δ*Fgatg1*, Δ*Fgtrs85*, Δ*Fgtrs85*Δ*Fgatg1* and Δ*Fgtrs85*Δ*TRAPPC11* strains expressing both FgAtg9-mNeonGreen and RFP-FgApe1 were examined by fluorescence microscopy under CM or MM-N medium. Bar = 10 μm. **(D)** The colocalization of FgAtg9-mNeonGreen with RFP-FgApe1 was quantified from panel C, with more than 300 FgAtg9-mNeonGreen puncta examined per strain. Error bars represent SD. Results represent three independent experiments. **P < 0.01; N.S., no significance.

To further define the role of TRAPPIII in AP formation in *F. graminearum*, we investigated its involvement in FgAtg9 trafficking. Atg9 is a conserved core autophagy protein that cycles between the PAS and various organelles to supply membrane material essential for AP biogenesis [18,19]. Affinity capture mass spectrometry identified FgTrs85 as a potential interacting partner of FgAtg9 (S3 Table). Subsequent GST pull-down and Y2H assays confirmed direct interactions between FgAtg9 and two TRAPPIII-specific subunits, FgTrs85 and TRAPPC13, while no interactions were observed with TRAPPC11 or TRAPPC12 (Fig 6A and 6B). To assess whether TRAPPIII regulates FgAtg9 transport in hyphae, we employed the TAKA (Transport of Atg9 after Knocking out *ATG1*) assay. In PH-1, FgAtg9 localized to multiple puncta, including the PAS, Golgi, mitochondria, and endosomes (Fig 6C and 6D). In the Δ*Fgatg1* mutant, which lacks retrograde FgAtg9 transport, FgAtg9 accumulated exclusively at the PAS under both nutrient-rich and starvation conditions, thereby validating the assay. In the Δ*Fgtrs85* mutant, FgAtg9 was distributed between the PAS and peripheral pools similar to PH-1, suggesting that FgTrs85 is dispensable for basal Atg9 cycling. However, in the Δ*Fgtrs85*Δ*Fgatg1* mutant, FgAtg9 remained in peripheral pools under nutrient-rich conditions but was restricted to the PAS during starvation, suggesting that FgTrs85 specifically promotes anterograde FgAtg9 delivery to the PAS under autophagy-inducing conditions. In contrast, in the Δ*Fgtrs85*Δ*trappc11* mutant, FgAtg9 failed to localize to the PAS under all conditions, indicating that the complete TRAPPIII complex is required for Atg9 recruitment to the PAS. These findings demonstrate that TRAPPIII plays a critical role in AP biogenesis by mediating the anterograde trafficking of FgAtg9 to the PAS, particularly under conditions that induce autophagy.

### TRAPPIII complex is required for intracellular transport in *F. graminearum*

**Fig 7:**
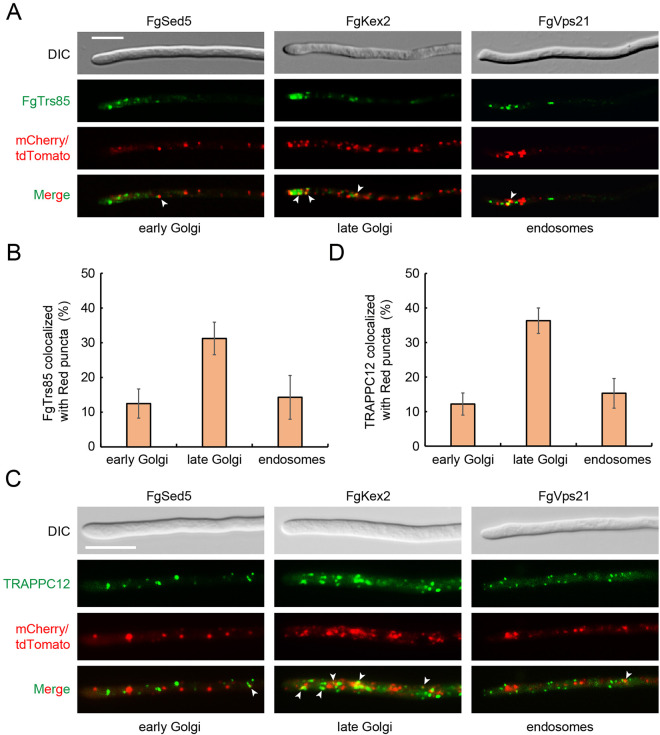
TRAPPIII complex localizes to the late Gogli of *F. graminearum.* **(A-D)** FgTrs85 and TRAPPC12 localize to the late Golgi in vegetative hyphae. PH-1 strains expressing either FgTrs85-mNeoGreen or TRAPPC12-GFP, along with mCherry-FgSed5, mCherry-FgSft2, or tdTomato-FgVps21 were examined by fluorescence microscopy in CM medium. Bar = 10 μm. Results from panel A or C were quantified of the co-localization of FgTrs85-mNeoGreen with the red dots, using the green dots for analysis. More than 50 hyphae were examined; the error bars in the graph represent the SD for the data from three independent experiments.

**Fig 8:**
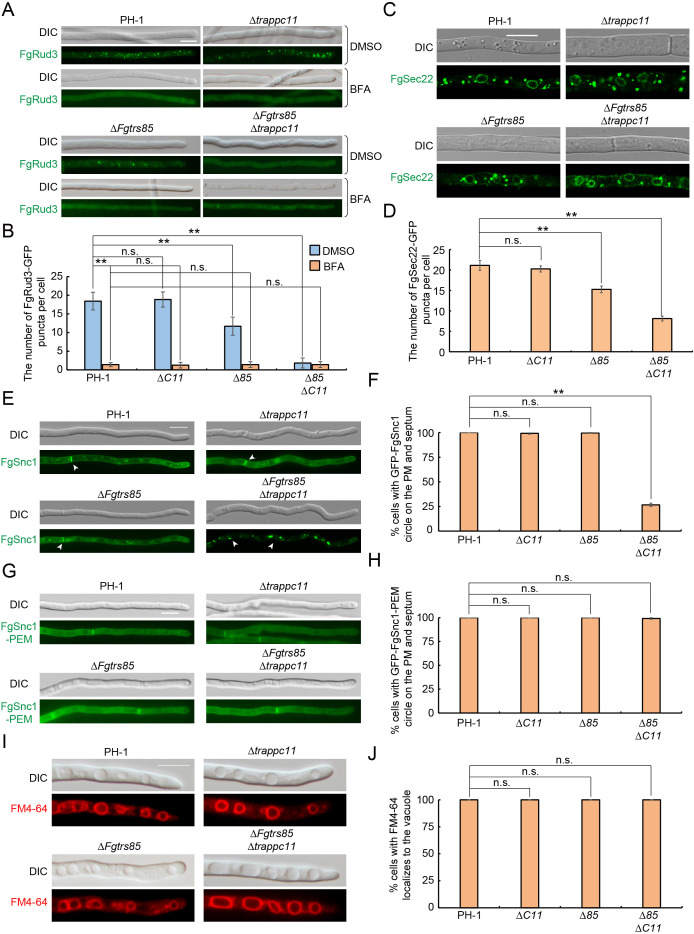
TRAPPIII complex is involved in intracellular transport of *F. graminearum.* **(A-B)** TRAPPIII is involved in the transport of FgRud3 to early Golgi. The PH-1, Δ*Fgatg1*, Δ*Fgtrs85*, and Δ*Fgtrs85*Δ*TRAPPC11* strains expressing FgRud3-GFP were treated with or without BFA, an inhibitor of ER-to-Golgi transport. Bar = 10 μm. Quantification of the number of FgRud3-GFP dots in each strain from panel A, and more than 300 cells in each strain were examined. **(C-D)** TRAPPIII is involved in the transport of FgSec22 to the early Golgi. The PH-1, Δ*Fgatg1*, Δ*Fgtrs85*, and Δ*Fgtrs85*Δ*TRAPPC11* strains expressing FgSec22-GFP were examined by fluorescence microscopy in CM medium. Bar = 10 μm. The number of FgSec22-GFP dots was quantified by examining more than 300 cells per strain. **(E-H)** TRAPPIII is involved in the transport of FgSnc1 from endosome to late-Golgi. PH-1, Δ*Fgatg1*, Δ*Fgtrs85*, and Δ*Fgtrs85*Δ*TRAPPC11* strains expressing GFP-FgSnc1 or GFP-FgSnc1-PEM were examined by fluorescence microscopy under CM medium. Bar = 10 μm. The localization of GFP-FgSnc1 or GFP-FgSnc1-PEM with PM and septum were quantified from panel E or G, and more than 100 cells in each strain were examined. **(I-J)** TRAPPIII is not involved in the endocytic pathway. FM4-64 staining of the vacuolar membrane of each strain was examined by fluorescence microscopy under CM medium. Error bars represent SD. Results represent three independent experiments. **P < 0.01; N.S., no significance.

As shown in Fig 2A, the Δ*Fgtrs85*Δ*trappc11* mutant exhibited more severe growth defects than the autophagy-deficient Δ*Fgatg1* mutant, suggesting additional roles for TRAPPIII in fungal physiology beyond autophagy. Given the critical role of intracellular transport in filamentous fungal growth [38], we investigated whether TRAPPIII influences hyphal development through effects on membrane trafficking. Localization studies showed that TRAPPIII-specific subunits FgTrs85 and TRAPPC12 predominantly reside at the late Golgi, with minor localization to the early Golgi and endosomes (Fig 7A–D). To evaluate TRAPPIII function in intracellular transport, we analyzed the localization of ER-to-early Golgi transport markers FgRud3 and FgSec22, which are crucial for the virulence and mycelial growth of *F. graminearum* [39]. We first examined the localization of FgRud3-GFP with or without Brefeldin A (BFA) treatment, which disrupts ER-to-Golgi transport. In contrast to the diffuse distribution of FgRud3-GFP observed in BFA-treated PH-1, both Δ*Fgtrs85* and Δ*Fgtrs85*Δ*trappc11* mutants showed markedly reduced puncta even in the absence of BFA treatment, indicating impaired ER-to-early Golgi transport. We further analyzed the localization of FgSec22, a v-SNARE that cycles between the ER and Golgi. Similarly, FgSec22 also exhibited significantly reduced puncta in Δ*Fgtrs85* and Δ*Fgtrs85*Δ*trappc11* mutants (Fig 8C and 8D), consistent with defective ER-to-early Golgi transport and reduced accumulation of FgSec22 at the early Golgi. To assess TRAPPIII’s involvement in plasma membrane (PM) recycling, we examined GFP-FgSnc1 [40], a v-SNARE required for the growth and pathogenicity of phytopathogenic fungus [41]. In PH-1 strains, GFP-FgSnc1 localized to the PM and septa, whereas in the Δ*Fgtrs85*Δ*trappc11* mutant, it was retained in cytoplasmic puncta (Fig 8E and 8F), suggesting a defect in either endosome-to-late Golgi or late Golgi-to-PM trafficking. To distinguish between these possibilities, we examined GFP-FgSnc1-PEM, a mutant variant that does not recycle via endocytosis [42]. GFP-FgSnc1-PEM localized normally to the PM and septa in all strains (Fig 8G and 8H), confirming that late Golgi-to-PM trafficking is intact. Thus, the defect in Δ*Fgtrs85*Δ*trappc11* specifically lies in endosome-to-late Golgi transport. Finally, FM4–64 staining showed normal endocytic trafficking to vacuoles across all tested strains (Fig 8I and 8J), indicating that TRAPPIII is not required for bulk endocytosis. In summary, TRAPPIII supports fungal growth and pathogenicity by regulating key intracellular trafficking steps, including ER-to-early Golgi and endosome-to-late Golgi transport, in addition to its essential role in autophagy.

### Genetic relationship between FgRab1 and the TRAPPIII complex

**Fig 9:**
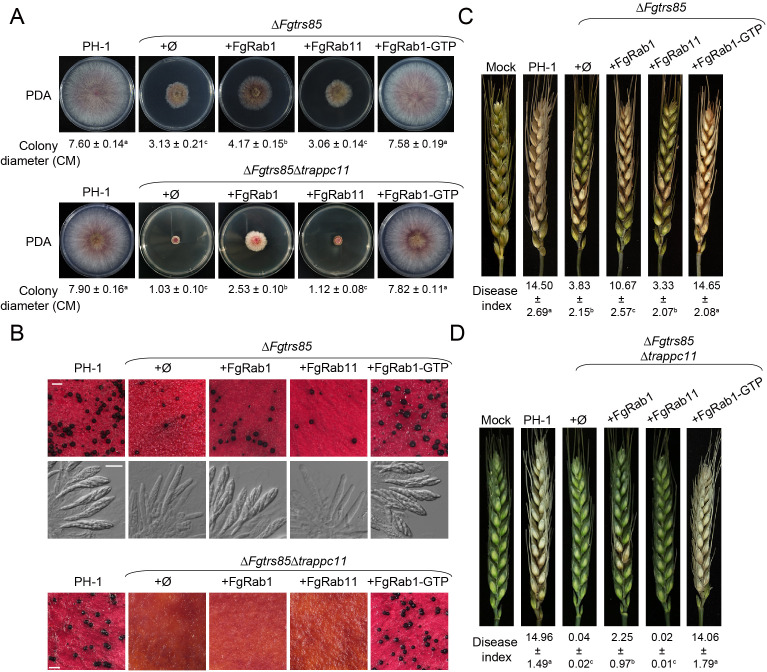
The growth and virulence defects of the Δ*Fgtrs85*, and Δ*Fgtrs85*Δ*TRAPPC11* strains can be suppressed by FgRab1. **(A)** The growth defect of Δ*Fgtrs85*, and Δ*Fgtrs85*Δ*TRAPPC11* mutant strains is suppressed by overexpression of FgRab1 and FgRab1-GTP, but not FgRab11. The PH-1, Δ*Fgtrs85*, Δ*Fgtrs85*Δ*Fgatg1* and the mutant strains transformed with plasmids for FgRab1, FgRab1-GTP or FgRab11 overexpression were determined on PDA plates. Colony diameters in each strain are shown under the growth image. **(B)** Overexpression of FgRab1-GTP can suppressed the defect of sexual reproduction of the Δ*Fgtrs85* and Δ*Fgtrs85*Δ*Fgatg1* mutant. PH-1, Δ*Fgtrs85*, Δ*Fgtrs85*Δ*Fgatg1* and the mutant strains transformed with plasmids for FgRab1, FgRab1-GTP or FgRab11 overexpression were determined on carrot agar plates. **(C-D)** Virulence defects of Δ*Fgtrs85*, and Δ*Fgtrs85*Δ*TRAPPC11* mutants are rescued by overexpression of FgRab1, but not FgRab11. Inoculation of conidial suspensions from the indicated strains into flowering wheat heads is shown, with the disease index for each strain indicated below the growth image. ± represent SD. The same letters indicate no statistical significance, while different letters indicate significant difference (p < 0.01).

In eukaryotes, TRAPPIII functions as a GEF that activates the small GTPase Rab1/Ypt1 to regulate autophagy [33,43]. To investigate the relationship between TRAPPIII and Rab1 in *F. graminearum*, we examined the interactions of TRAPPIII-specific subunits with FgRab1. Our results demonstrated that FgTrs85 and TRAPPC12 interact with FgRab1, as evidenced by Y2H and GST pull-down assays (S10 Fig). Next, we tested whether perturbing FgRab1 activity phenocopies the effects of TRAPPIII-specific subunit deletions. Given *FgRAB1* is essential for viability, we generated a hypomorphic allele, *Fgrab1–2*, by substituting the conserved glycine at position 83 with an alanine. The *Fgrab1–2* mutant exhibited severe defects in mycelial growth, perithecium formation, and virulence (S11A, S11B and S11C Fig), as well as impaired autophagy and disrupted ER-to-early Golgi and endosome-to-late Golgi transport (S12A–I Fig). These phenotypes closely resembled those of the Δ*Fgtrs85*Δ*trappc11* mutant, suggesting that FgRab1 and TRAPPIII act in the same genetic pathway regulating fungal development and virulence. To determine whether TRAPPIII contributes to these processes by activating FgRab1, we overexpressed either FgRab1 or FgRab11 in the Δ*Fgtrs85* and Δ*Fgtrs85*Δ*trappc11* mutants. Overexpression of FgRab1, but not FgRab11, partially rescued defects in mycelial growth, ascospore production, and virulence in the Δ*Fgtrs85* mutant (Fig 9A, 9B and 9C). However, overexpression of a constitutively active, GTP-bound form of FgRab1 fully rescued these defects (Fig 9A, 9B and 9C). In the background of the Δ*Fgtrs85*Δ*trappc*11 mutant, FgRab1 overexpression improved growth and virulence, although it did not enhance perithecium formation (Fig 9A, 9B, and 9D). Strikingly, overexpression of FgRab1-GTP fully restored normal growth, perithecium development, and virulence in the Δ*Fgtrs85*Δ*trappc11* mutant (Fig 9A, 9B, and 9D). Subsequently, we tested whether FgRab1 activation could also rescue autophagy defects. In starved PH-1 cells treated with PMSF, autophagic bodies accumulated in vacuoles, whereas no such accumulation was observed in the autophagy-deficient Δ*Fgatg1* or Δ*Fgtrs85*Δ*trappc11* mutants (S13 Fig) [44]. Notably, overexpression of FgRab1-GTP, but not wild-type FgRab1, restored autophagic body accumulation in Δ*Fgtrs85*Δ*trappc11*, indicating recovery of autophagy, consistent with the restoration of perithecium formation. Collectively, these genetic results demonstrate that TRAPPIII regulates growth, development, and virulence in *F. graminearum* primarily by activating the small GTPase FgRab1.

## Discussion

**Fig 10:**
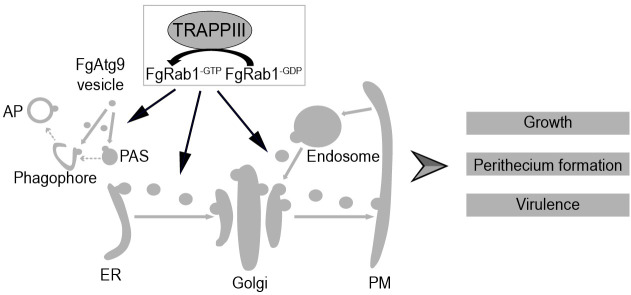
Diagram of TRAPPIII complex-mediated regulation of growth, perithecium formation, and virulence in *F*. *graminearum.*

Understanding the pathogenic mechanisms of *F. graminearum*, the causal agent of wheat head blight, is crucial for developing targeted control strategies. In this study, we identified and characterized the TRAPPIII complex in *F. graminearum*, which comprises four specific subunits (FgTrs85, TRAPP11, TRAPP12, and TRAPP13) and demonstrated that this complex is essential for fungal growth, development, and virulence. Functionally, TRAPPIII localizes to the PAS during autophagy, where it recruits FgAtg9 to mediate AP biogenesis (Fig 10), a process crucial for both perithecium formation and virulence. In addition, TRAPPIII also localizes to the Golgi and thereby regulates two key intracellular transport pathways: ER-to-early Golgi and endosome-to-late Golgi (Fig 10), thereby ensuring proper distribution of FgSec22, FgRud3, and FgSnc1, which are required for sustained hyphal growth and virulence. Collectively, these findings establish TRAPPIII as a central regulator of intracellular transport and autophagy that governs fungal development and virulence.

The TRAPPIII complex was first identified in the *S. cerevisiae*, where much of our foundational understanding of its structure and function was developed [33,45,46]. However, significant differences exist between yeast and filamentous fungi. In *F. graminearum*, the TRAPPIII complex comprises the conserved FgTrs85 subunit along with three metazoan-like subunits: TRAPPC11, TRAPPC12, and TRAPPC13, which are absent in yeast but conserved in higher eukaryotes. A similar composition has been reported in *Aspergillus nidulans* [47], suggesting that filamentous fungal TRAPPIII complexes are more closely related to their metazoan counterparts than to the yeast complex. Although TRAPPC11, TRAPPC12, and TRAPPC13 are functionally important in metazoans, for example, TRAPPC11 is essential for growth [48], TRAPPC12 regulates oligodendrocyte differentiation [49], and TRAPPC13 contributes to the Golgi stress response [50]. Complete deletion of these subunits in *F. graminearum* does not impair growth, development, or virulence (S3 Fig). In contrast, FgTrs85 is not essential, similar to *S. cerevisiae* [34], but its combined deletion with any of the other TRAPPIII-specific subunits results in severe defects, underscoring a level of functional redundancy. These findings support the notion that TRAPPIII in filamentous fungi represents a functional intermediate between yeast and metazoan complexes.

Autophagy is a conserved catabolic pathway essential for maintaining intracellular homeostasis and plays crucial roles in perithecium development and plant infection in *F. graminearum* [10]. While TRAPPIII was originally implicated as an autophagy regulator in *S. cerevisiae*, our study confirms its similar role in *F. graminearum*, albeit with notable distinctions. The four TRAPPIII-specific subunits display different contributions to autophagy. For instance, unlike in yeast or mammals, where the deletion of Trs85/TRAPPC8 fully blocks autophagy, the deletion of Δ*Fgtrs85* in *F. graminearum* only partially impairs this process (Figs 3A, 3B, 3C, 5A and 5B), thus allowing for perithecium formation. In contrast, double mutants lacking FgTrs85 and another TRAPPIII-specific subunit demonstrate a complete blockade of autophagy and are unable to form perithecia (Figs 3A, 3B and 2C), indicating that these subunits function synergistically to regulate autophagy.

AP formation requires the membrane protein Atg9, a central player in PAS initiation [17]. In *M. oryzae*, the phosphorylation of MoAtg9 by MoMkk1 regulates AP biogenesis and pathogenicity [51]. In *F. graminearum*, the trafficking of FgAtg9 depends on the small GTPase FgRab7 [26]. Our results further demonstrate that TRAPPIII, through its subunits FgTrs85 and TRAPPC13, interacts with FgAtg9 to regulate its anterograde transport to the PAS, where TRAPPIII localizes exclusively, in a pattern reminiscent of other Atg proteins (e.g., Atg2, Atg5) involved in AP formation [37]. Given the well-established role of TRAPP as a tethering complex, we speculate that TRAPPIII facilitates the vesicle tethering of FgAtg9 at the PAS, thereby initiating APs biogenesis.

Efficient intracellular transport is vital for fungal growth and host invasion [38]. A key divergence between *F. graminearum* and *S. cerevisiae* lies in the functional importance of TRAPPIII: while its loss has negligible impact on yeast growth, it causes severe defects in *F. graminearum*. This discrepancy likely arises because TRAPPIII in *F. graminearum* has essential roles beyond autophagy, especially in intracellular transport. Supporting this, TRAPPIII deletion leads to more severe growth impairment than Δ*Fgatg1* mutant, indicating that defects stem from disrupted transport rather than autophagy alone. Specifically, we show that TRAPPIII is required for maintaining the localization of FgSec22 and FgRud3 to the early Golgi, implicating it in ER-to-Golgi trafficking. In *S. cerevisiae*, TRAPPI partially compensates for TRAPPIII function; however, no such redundancy exists in *F. graminearum*, which explains the severe phenotypes observed. Additionally, TRAPPIII regulates endosome-to-late Golgi trafficking of FgSnc1, further linking it to growth and virulence. Crucially, we identified FgRab1 as a key downstream target of TRAPPIII, with constitutively active FgRab1 (GTP-bound) fully rescuing the Δ*Fgtrs85*Δ*trappc11* mutant phenotypes. Although TRAPPII was previously suggested to activate Ypt1 in *S. cerevisiae* for endosome-to-late Golgi transport [52], our genetic data are consistent with TRAPPIII being a GEF for FgRab1 in *F. graminearum*, coordinating both ER-to-early Golgi and endosome-to-late Golgi transport pathways. These findings align with recent reports that support a conserved role of TRAPPIII in Rab1 activation [45].

The TRAPP complex is currently the only known large tethering complex that possesses intrinsic GEF activity. Our study firmly establishes TRAPPIII as a master regulator of multiple physiological processes in *F. graminearum* through the activation of the small GTPase FgRab1. The complete rescue of TRAPPIII-deficient mutants via overexpression of FgRab1-GTP highlights the centrality of this regulatory axis. This conserved GEF-Rab module integrates autophagy and intracellular transport to control fungal development and virulence. Given the broad regulatory scope of FgRab1, future studies should focus on identifying its specific effectors at distinct regulatory levels and characterizing their functions. Such investigations will yield critical insights into the molecular underpinnings of *F. graminearum* growth, development, and virulence.

## Materials and methods

### *F. graminearum* strains and culture conditions

The fungal strains utilized in this study are detailed in S1 Table. Gene deletion and transformations of *F. graminearum* were conducted as previously described [12].

The mycelial growth assay was performed as previously described [39]. Briefly, small agar blocks containing mycelia were excised from the edge of 3-day-old cultures and placed onto fresh potato dextrose agar (PDA), followed by incubation at 25°C for 3 days. For live-cell fluorescence microscopy, fresh mycelia from each strain were inoculated in complete medium (CM) at 25°C for 16 h. Autophagy was induced by transferring mycelia of each strain from CM to minimal media without nitrogen (MM-N) for 6 h of nitrogen-starvation at 25°C. To stain the vacuole, 1.0 μM CMAC was added to the harvested hyphae for 10 min [11]. FM4–64 staining was used to detect fungal endocytic transport as described previously [53]. For conidia production, each strain was inoculated in carboxymethyl cellulose (CMC) liquid medium at 25°C for 5 days [39]. For perithecia production, all strains were inoculated in carrot agar plates at 25°C and assayed as described previously [40].

### Virulence assay

The virulence assay conducted on the infection of flowering wheat heads was performed as described previously [12]. Briefly, *Triticum aestivum* L. cv. Jimai 22 was used for the virulence assay. Conidia harvested from 5-day-old CMC medium were resuspended in sterile distilled water to achieve a concentration of 4 × 10^5^ spores/ml. A 10 μl aliquot of the conidial suspension was inoculated into a floret of the flowering wheat head. Symptomatic spikelets on the wheat spikes were assessed and photographed at 14 days post-inoculation (dpi).

### Deoxynivalenol (DON) production and quantitative RT-PCR analysis

The production of DON and the quantitative analysis of *TRI* gene expression were conducted as previously described [12]. Detailed information regarding the primers used for quantitative reverse transcription polymerase chain reaction (qRT-PCR) analysis are provided in S2 Table.

### Fluorescence microscopy

For live-cell fluorescence microscopy, hyphae expressing fluorescent proteins or stained with fluorescent dyes (FM4–64 or CMAC) were visualized using an upright research microscope (Eclipse Ni-U; Nikon). Images were acquired and combined as previously described [40]. The quantification of fluorescence images was performed as specified in the figure legends.

### Y2H assay

The Y2H assay was used to detect protein-protein interactions using the Matchmaker GAL4 two-hybrid system 3 (Clontech). Full-length cDNAs of TRAPPIII-specific subunits were constructed into pGBKT7 or pGADT7 vectors as bait and prey constructs, respectively. The primers utilized for gene cloning are listed in S2 Table. The yeast strain AH109 was co-transformed with the prey and bait constructs and selected on synthetic dextrose (SD)-Leu-Trp agar plates. Yeast cells were cultured overnight at 26°C in SD-Leu-Trp medium before being spotted onto agar plates. Growth of yeast cells was conducted on SD-Leu-Trp agar plates, while interactions were assessed on SD-Leu-Trp-His-Ade agar plates. The pGBKT7 and pGADT7 plasmids served as negative controls for interaction.

### GST pull-down and Co-IP assay

For GST pull-down assay, TRAPPIII-specific subunits fused with GST or His tags were constructed in the pET41 and pET30 vectors, respectively. The GST-fusion or His-fusion proteins of TRAPPIII-specific subunits were transformed into the *Escherichia coli* strain BL21 and purified using conventional methods [54]. GST pull-down was performed with a GST-tag Protein Purification Kit (Beyotime, Biyuntian Biotech) as previously described [55]. Subsequently, the samples were analyzed by immunoblotting with anti-GST or anti-His antibodies. For Co-IP assay, FgTrs85 was fused with GFP at its N-terminus and cloned into the pYF11 vector, while TRAPPC11/12/13 were fused with a His tag at their C-termini and constructed into the pHD64 vector. The GFP-FgTrs85 and TRAPPC11/12/13-His constructs were then co-transformed into the PH-1 strain. Total proteins extract from positive transformants were incubated with anti-GFP beads. The bead-bound samples were subsequently eluted and analyzed by immunoblotting using anti-GFP, anti-His, and anti-GAPDH antibodies.

### Protease protection assay

For the protease-protection assay, fresh mycelia of each strain expressing GFP-FgAtg8 were inoculated in CM medium for 16 h and then shifted to MM-N for 5 h of starvation. The mycelia were washed with DTT buffer and subsequently lysed to release protoplasts. Unbroken mycelia were removed by centrifugation at 2,000 × g for 5 min, and washed with SP buffer. The samples were centrifuged at 10,000 × g for 10 min and the membrane components were precipitated by resuspension in PS200 buffer. The PS200 buffer, containing membrane components, was treated or untreated with PK (200 μg/ml) and/or Triton X-100 (TX) as previously described (Nair et al. 2011). Following this, the samples were treated with TCA and washed with acetone. The presence of mature APs in different strains was analyzed by immunoblotting using a GFP antibody as described previously [39].

### Immunoblot assay

Immunoblotting was performed using the indicated antibodies and subjected to ECL HRP substrate (P90720; Millipore) as previously described [39]. Blots were immunoblotted with anti-GST, anti-His or anti-GFP (TransGen Biotech, China), and with Ponceau staining used as a control. The density of the immunoblot bands was quantified using ImageJ software (National Institutes of Health, US).

### Statistical analyses

All experimental data were obtained from three independent replicates to ensure reproducibility of the observed trends. The statistical significance of differences between group means was assessed using ANOVA, while paired-sample comparisons were performed using Student’s t-test, as previously described [12].

## Supporting information

S1 Fig Phylcogenetic analysis of *F. graminearum* TRAPPIII-specific subunits and its orthologs. The GenBank accession numbers of the sequences are as follows: OAO93538.1 (*Arabidopsis thaliana* Trs85), OAO90306.1 (*Arabidopsis thaliana* TRAPPC11), NP_566117.1 (*Arabidopsis thaliana* TRAPPC13), KAH7214901.1 (*Fusarium oxysporum* Trs85), EXA41198.1(*Fusarium oxysporum* TRAPPC11), EWZ96067.1 (*Fusarium oxysporum* TRAPPC12), EXA42516.1 (*Fusarium oxysporum* TRAPPC13), XP_962212.2 (*Neurospora crassa* Trs85), KAK3504041.1 (*Neurospora crassa* TRAPPC11), XP_011394108.1 (*Neurospora crassa* TRAPPC12), XP_956870.3 (*Neurospora crassa* TRAPPC13), XP_003715283.1 (*Magnaporthe oryzae* Trs85), KAH8840300.1 (*Magnaporthe oryzae* TRAPPC11), KAH8841361.1 (*Magnaporthe oryzae* TRAPPC12), XP_003717829.1 (*Magnaporthe oryzae* TRAPPC13), XP_006722483.1 (*Homo sapiens* Trs85/TRAPPC8), NP_068761.4 (*Homo sapiens* TRAPPC11), XP_011508652.1 (*Homo sapiens* TRAPPC12), NP_001087224.1 (*Homo sapiens* TRAPPC13), XP_680580.1 (*Aspergillus nidulans* Trs85), XP_658978.1 (*Aspergillus nidulans* TRAPPC11), XP_050467586.1 (*Aspergillus nidulans* TRAPPC12), XP_661962.1 (*Aspergillus nidulans* TRAPPC13), CAD6609984.1 (*Saccharomyces cerevisia*e Trs85), XP_044399494.1 (*Triticum aestivum* Trs85), XP_044413823.1 (*Triticum aestivum* TRAPPC11), XP_044399151.1 (*Triticum aestivum* TRAPPC13), XP_011390182.1 (*Ustilago maydi*s Trs85), XP_011390925.1 (*Ustilago maydi*s TRAPPC11), XP_011390267.1 (*Ustilago maydi*s TRAPPC13), NP_647785.3 (*Drosophila melanogaster* TRAPPC11), XP_001360082.3 (*Drosophila melanogaster* TRAPPC12), NP_609365.3 (*Drosophila melanogaster* TRAPPC13). The protein sequences were aligned using the CLUSTALW program and the phylogenetic tree was generated by MEGA 7.0.
(TIF)

S2 Fig FgTrs85 interacts with TRAPPC11, TRAPPC12, and TRAPPC13 *in vivo.* Co-IP assays were performed using the PH-1 strain. Total protein lysates from strains co-expressing GFP-FgTrs85 and TRAPPC11/12/13-His were incubated with anti-GFP beads. The immunoprecipitated samples and input controls were analyzed by immunoblotting with the indicated antibodies. Anti-GAPDH antibody was used as a loading control for the input lysates.
(TIF)

S3 Fig Construction of TRAPPIII-specific subunits mutant strains. (A) Schematic of TRAPPIII-specific subunits deletion strategy in *F. graminearum*. The *FgTRS85*, *TRAPPC11*, *TRAPPC12* or *TRAPPC13* gene was replaced by hygromycin (*HPH*) cassette to construct single-gene knockout mutants. In the Δ*Fgtrs85* mutant, *TRAPPC11*, *TRAPPC12* or *TRAPPC13* gene was replaced with nourseothricin (*NAT*) cassette to construct double knockout mutant strains. (B-C) Identification of the mutant strains. Detection of target gene knockout in transformants using diagnostic PCR.
(TIF)

S4 Fig Δ*TRAPPC11*Δ*TRAPPC12*Δ*TRAPPC13* do not affect both growth and virulence in *F. graminearum.* (A-B) Schematic of the Δ*TRAPPC11*Δ*TRAPPC12*Δ*TRAPPC13* mutant construction strategy in *F. graminearum.* The *TRAPPC11*, *TRAPPC12* and *TRAPPC13* gene were replaced by *HPH*, *NAT* and *KAN* cassette, respectively. Diagnostic PCR was employed to confirm the knockout of the target genes in the transformants. (C-D) The Δ*TRAPPC11*Δ*TRAPPC12*Δ*TRAPPC13* mutant strains exhibit normal vegetative growth and virulence. PH-1 and mutant strains were cultured on PDA for 3 days; colony diameters in each strain are shown under the growth image. Inoculation of conidial suspensions of PH-1 and mutant strains into flowering wheat heads; disease index in each strain is shown under the growth image.
(TIF)

S5 Fig Complementation with FgTrs85 restores growth in mutants lacking TRAPPIII-specific subunits. The colony growth and corresponding diameter measurements of the indicated strains were recorded after 3 days of culture on PDA. Data are from three independent experiments. ± represent SD. Different lowercase letters above the bars denote statistically significant differences (p < 0.01).
(TIF)

S6 Fig TRAPPIII complex is involved in DON biosynthesis of *F. graminearum.* (A-B) Defective DON biosynthesis in TRAPPIII-specific subunits mutants. Levels of DON production were determined in wheat seeds infected with PH-1 and TRAPPIII-specific subunits mutant strains. ± represent SD. The same letters indicate no statistical significance, while different letters indicate significant difference (p < 0.01). The PH-1 and TRAPPIII-specific subunits mutant strains expressing Tri4-GFP were visualized by fluorescence microscopy under both CM and TBI medium.
(TIF)

S7 Fig TRAPPIII complex is involved in asexual reproduction of *F. graminearum.* (A) TRAPPIII is involved in conidia production. PH-1 and mutant strains were cultured in CMC medium for 5 days to produce conidia. (B-D) TRAPPIII is involved in the germination of conidia. Conidia of PH-1 and mutant strains were incubated in liquid YEPD medium for 0 and 4 hours and conidial germination was detected by using live-cell microscopy. Conidial length and germination rates were quantified. ± represent SD. The same letters indicate no statistical significance, while different letters indicate significant difference (p < 0.01).
(TIF)

S8 Fig TRAPPC12 fails to localize to the PAS in the Δ*Fgatg1*Δ*trappc13* mutant. (A) The localization of TRAPPC12 to the PAS was examined in Δ*Fgatg1* and Δ*Fgatg1*Δ*trappc13* mutants. The strains co-expressing TRAPPC12-GFP and RFP-FgApe1 were examined by fluorescence microscopy under CM or MM-N conditions. Arrowheads indicate the PAS, marked by RFP-FgApe1 puncta, that colocalize with TRAPPC12-GFP. Bar = 10 μm. (B) The quantification of colocalization between RFP-FgApe1 and TRAPPC12-GFP from panel A is presented. More than 300 RFP-FgApe1 puncta were examined for each strain. Error bars represent SD. Results represent three independent experiments. **P < 0.01.
(TIF)

S9 Fig FgTrs85 localizes to the PAS of *F. graminearum.* (A) The localization of FgTrs85 to the PAS in Δ*Fgatg1* and Δ*Fgatg1*Δ*trappc12* mutants was assessed. The indicated strains co-expressing FgTrs85-mNeoGreen and RFP-FgApe1 were examined by fluorescence microscopy under CM or MM-N conditions. Arrowheads indicate RFP-FgApe1 puncta that colocalize with FgTrs85-mNeoGreen. Bar = 10 μm. (B) Quantification of the colocalization between RFP-FgApe1 and FgTrs85-mNeoGreen from panel A. More than 300 RFP-FgApe1 puncta were examined for each strain. Error bars represent SD. Results represent three independent experiments. **P < 0.01.
(TIF)

S10 Fig FgRab1 interacts with TRAPPIII-specific subunits in *F. graminearum.* (A) FgRab1 interacts with FgTrs85 and TRAPPC12, but not with TRAPPC11 and TRAPPC13, in Y2H assay. Yeast cells were growth on agar plates of SD-Leu-Trp (growth) and SD-Leu-Trp-His-Ade (interaction). (B) A GST pull-down assay confirms the interaction between TRAPPIII-specific subunits and FgRab1 in vitro. GST-tagged FgTrs85, TRAPPC11, TRAPPC12 and TRAPPC13, along with His-tagged FgRab1, were expressed in *E. coli*. Western blot was performed using antibodies against GST and His.
(TIF)

S11 Fig FgRab1 is required for vegetative growth, perithecia formation and full virulence of *F. graminearum.* (A) The *Fgrab1–2* mutant exhibit vegetative growth defects. PH-1 and *Fgrab1–2* strains were cultured on PDA for 3 days. (B) The *Fgrab1–2* mutant exhibit perithecia formation defects during the sexual reproduction. PH-1 and *Fgrab1–2* mutant strains were cultured on carrot agar plates to detect perithecia production. (C) The *Fgrab1–2* mutant exhibit virulence defects under plant infection. Conidial suspensions of the PH-1 and mutant strains were inoculated into flowering wheat heads.
(TIF)

S12 Fig FgRab1 is required for autophagy and intracellular transport. (A) The *Fgrab1–2* mutant is defective in the transport of GFP-FgAtg8 to the vacuole. Hyphae of PH-1 and *Fgrab1–2* expressing GFP-FgAtg8 were examined by fluorescence microscopy after growth in CM or MM-N medium. Vacuoles were stained with CMAC before visualization by microscopy. Bar = 10 μm. (B) Quantification of GFP-FgAtg8 transport to the vacuole from panel A. More than 200 cells were analyzed for each strain. Error bars represent SD. Results represent three independent experiments. **P < 0.01; N.S., no significance. (C) The *Fgrab1–2* mutant exhibits a defect in GFP-FgAtg8 processing under starvation. Hyphae of WT and mutant strains expressing GFP-FgAtg8 were grown in CM medium and then shifted to MM-N. Protein extracts were resolved in vegetative hyphae lysates by immunoblot analysis using anti-GFP antibodies; the percentage of free GFP in each lane is shown under the blot. (D-E) FgRab1 is involved in the transport of FgRud3 to early Golgi. PH-1 and *Fgrab1–2* strains expressing FgRud3-GFP were examined by fluorescence microscopy in CM medium. Bar = 10 μm. Quantification of the number of FgRud3-GFP dots in each strain from panel D, and more than 300 cells in each strain were examined. (F-I) FgRab1 is involved in the transport of FgSnc1 from endosome to late-Golgi. PH-1 and Fgrab1–2 strains expressing GFP-FgSnc1 or GFP-FgSnc1-PEM were examined by fluorescence microscopy in CM medium. Bar = 10 μm. The localization of GFP-FgSnc1 or GFP-FgSnc1-PEM with PM and septum were quantified from panel F or G, and more than 100 cells in each strain were examined.
(TIF)

S13 Fig Autophagy defects of Δ*Fgtrs85*Δ*TRAPPC11* can be suppressed by FgRab1-GTP, but not FgRab1-WT. PH-1, Δ*Fgatg1*, Δ*Fgtrs85*Δ*TRAPPC11*, and Δ*Fgtrs85*Δ*TRAPPC11* strains overexpressing FgRab1 or FgRab1-GTP were grown in CM medium and then shifted to MM-N containing 2 mM of PMSF. Overexpression of FgRab1-GTP in Δ*Fgtrs85*Δ*TRAPPC11* and PH-1 hyphae accumulate autophagic bodies inside their vacuole. Arrowheads indicate autophagic bodies in the vacuole of hyphae. Bar = 10 μm.
(TIF)

S1 Table *F. graminearum* strains used in this study. (DOCX)

S2 Table Primers used in this study. (DOCX)

S3 Table A list of putative FgTrs85-interacting proteins under starvation-induced condition identified by mass spectrometry assay. (DOCX)
