# Supplementary figures and images for: The TRAPPIII complex regulates development and virulence of Fusarium graminearum by coordinating autophagy and intracellular transport
Source: PLoS Pathog. 2025 Oct 24;21(10):e1013627. doi: 10.1371/journal.ppat.1013627 (PMC12578332; doi:10.1371/journal.ppat.1013627)

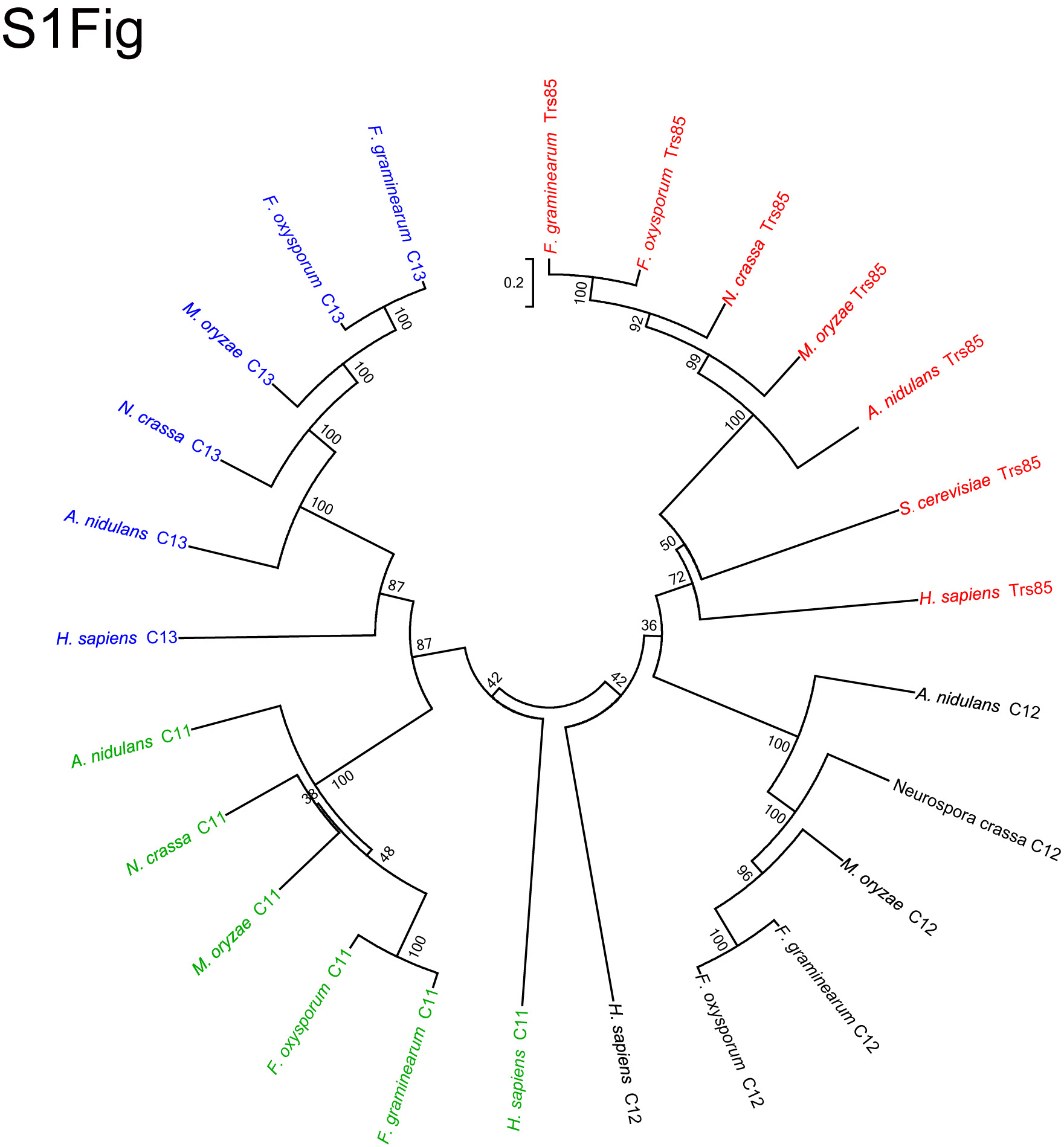

Supplement: S1 Fig — The GenBank accession numbers of the sequences are as follows: OAO93538.1 (Arabidopsis thaliana Trs85), OAO90306.1 (Arabidopsis thaliana TRAPPC11), NP_566117.1 (Arabidopsis thaliana TRAPPC13), KAH7214901.1 (Fusarium oxysporum Trs85), EXA41198.1(Fusarium oxysporum TRAPPC11), EWZ96067.1 (Fusarium oxysporum TRAPPC12), EXA42516.1 (Fusarium oxysporum TRAPPC13), XP_962212.2 (Neurospora crassa Trs85), KAK3504041.1 (Neurospora crassa TRAPPC11), XP_011394108.1 (Neurospora crassa TRAPPC12), XP_956870.3 (Neurospora crassa TRAPPC13), XP_003715283.1 (Magnaporthe oryzae Trs85), KAH8840300.1 (Magnaporthe oryzae TRAPPC11), KAH8841361.1 (Magnaporthe oryzae TRAPPC12), XP_003717829.1 (Magnaporthe oryzae TRAPPC13), XP_006722483.1 (Homo sapiens Trs85/TRAPPC8), NP_068761.4 (Homo sapiens TRAPPC11), XP_011508652.1 (Homo sapiens TRAPPC12), NP_001087224.1 (Homo sapiens TRAPPC13), XP_680580.1 (Aspergillus nidulans Trs85), XP_658978.1 (Aspergillus nidulans TRAPPC11), XP_050467586.1 (Aspergillus nidulans TRAPPC12), XP_661962.1 (Aspergillus nidulans TRAPPC13), CAD6609984.1 (Saccharomyces cerevisiae Trs85), XP_044399494.1 (Triticum aestivum Trs85), XP_044413823.1 (Triticum aestivum TRAPPC11), XP_044399151.1 (Triticum aestivum TRAPPC13), XP_011390182.1 (Ustilago maydis Trs85), XP_011390925.1 (Ustilago maydis TRAPPC11), XP_011390267.1 (Ustilago maydis TRAPPC13), NP_647785.3 (Drosophila melanogaster TRAPPC11), XP_001360082.3 (Drosophila melanogaster TRAPPC12), NP_609365.3 (Drosophila melanogaster TRAPPC13). The protein sequences were aligned using the CLUSTALW program and the phylogenetic tree was generated by MEGA 7.0. (TIF) [file ppat.1013627.s001.tif]

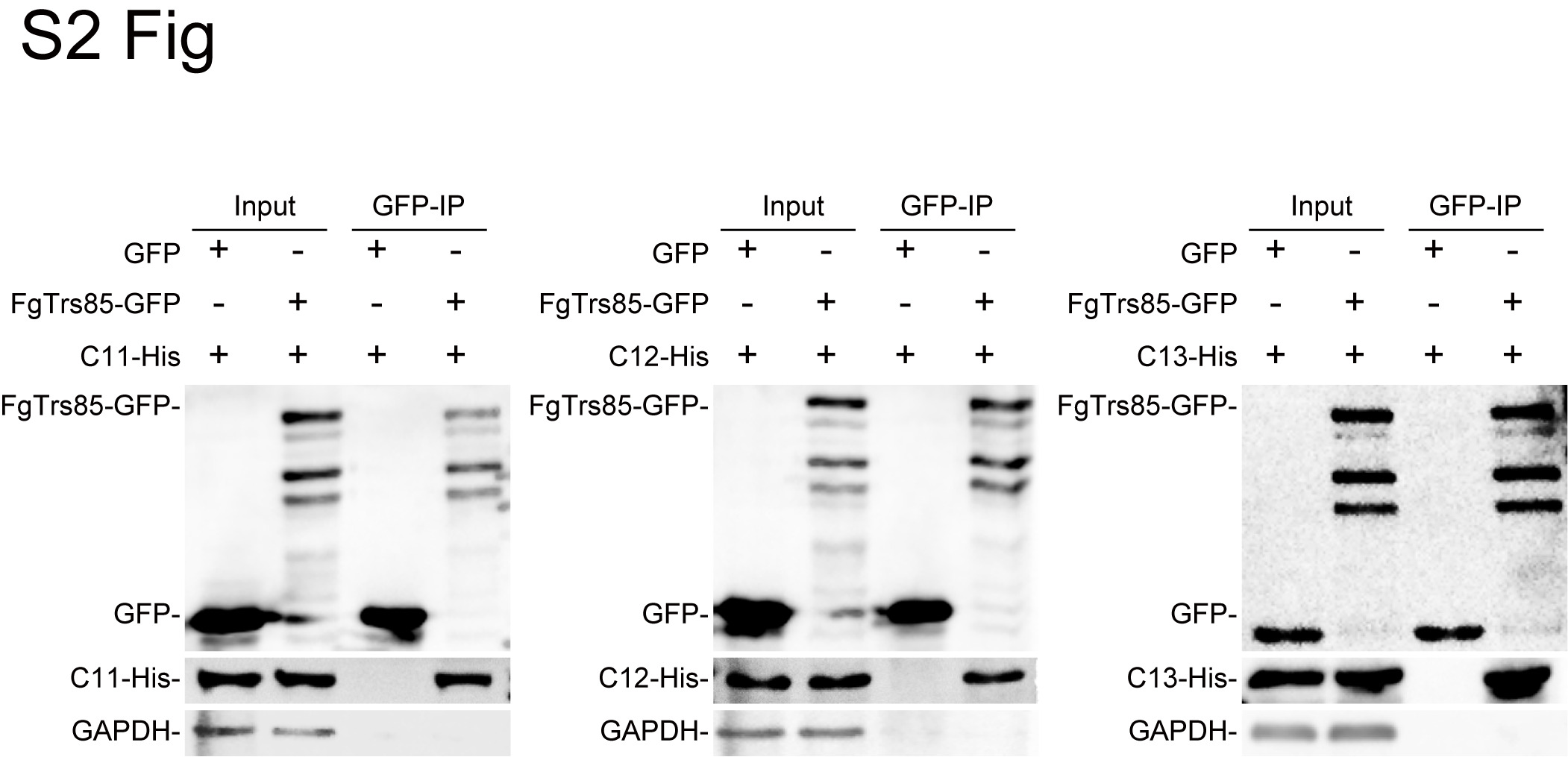

Supplement: S2 Fig — Co-IP assays were performed using the PH-1 strain. Total protein lysates from strains co-expressing GFP-FgTrs85 and TRAPPC11/12/13-His were incubated with anti-GFP beads. The immunoprecipitated samples and input controls were analyzed by immunoblotting with the indicated antibodies. Anti-GAPDH antibody was used as a loading control for the input lysates. (TIF) [file ppat.1013627.s002.tif]

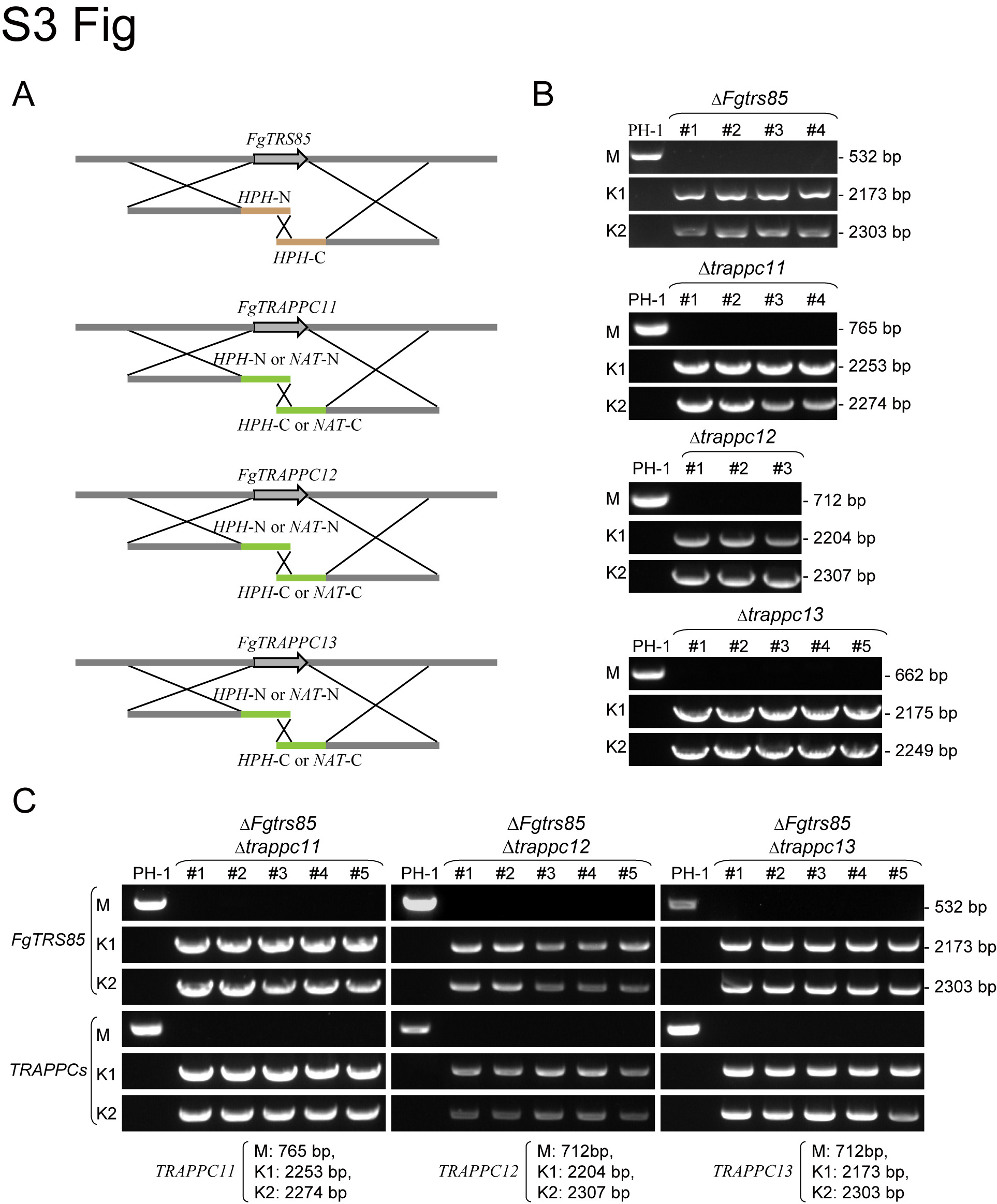

Supplement: S3 Fig — (A) Schematic of TRAPPIII-specific subunits deletion strategy in F. graminearum. The FgTRS85, TRAPPC11, TRAPPC12 or TRAPPC13 gene was replaced by hygromycin (HPH) cassette to construct single-gene knockout mutants. In the ΔFgtrs85 mutant, TRAPPC11, TRAPPC12 or TRAPPC13 gene was replaced with nourseothricin (NAT) cassette to construct double knockout mutant strains. (B-C) Identification of the mutant strains. Detection of target gene knockout in transformants using diagnostic PCR. (TIF) [file ppat.1013627.s003.tif]

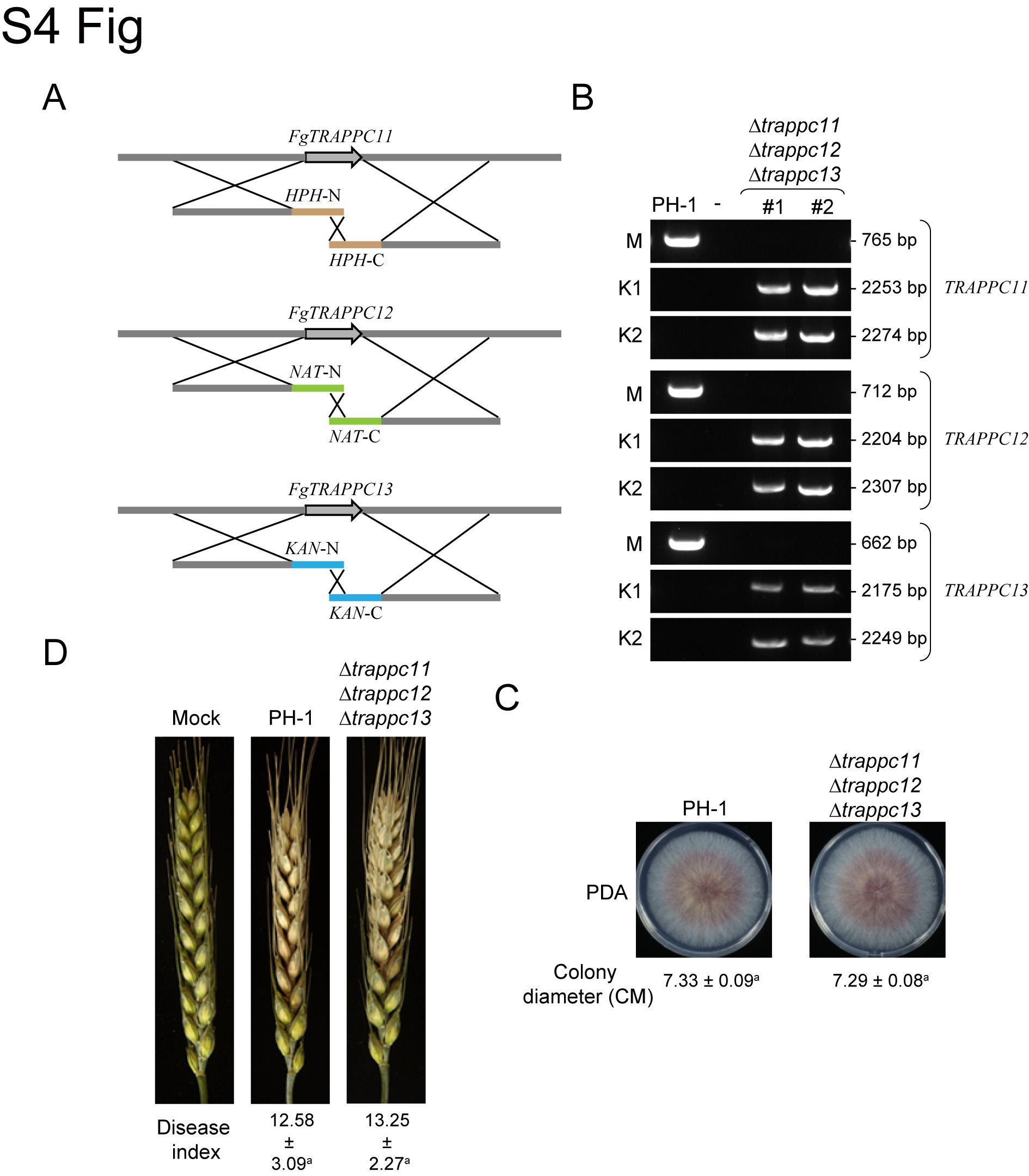

Supplement: S4 Fig — (A-B) Schematic of the ΔTRAPPC11ΔTRAPPC12ΔTRAPPC13 mutant construction strategy in F. graminearum. The TRAPPC11, TRAPPC12 and TRAPPC13 gene were replaced by HPH, NAT and KAN cassette, respectively. Diagnostic PCR was employed to confirm the knockout of the target genes in the transformants. (C-D) The ΔTRAPPC11ΔTRAPPC12ΔTRAPPC13 mutant strains exhibit normal vegetative growth and virulence. PH-1 and mutant strains were cultured on PDA for 3 days; colony diameters in each strain are shown under the growth image. Inoculation of conidial suspensions of PH-1 and mutant strains into flowering wheat heads; disease index in each strain is shown under the growth image. (TIF) [file ppat.1013627.s004.tif]

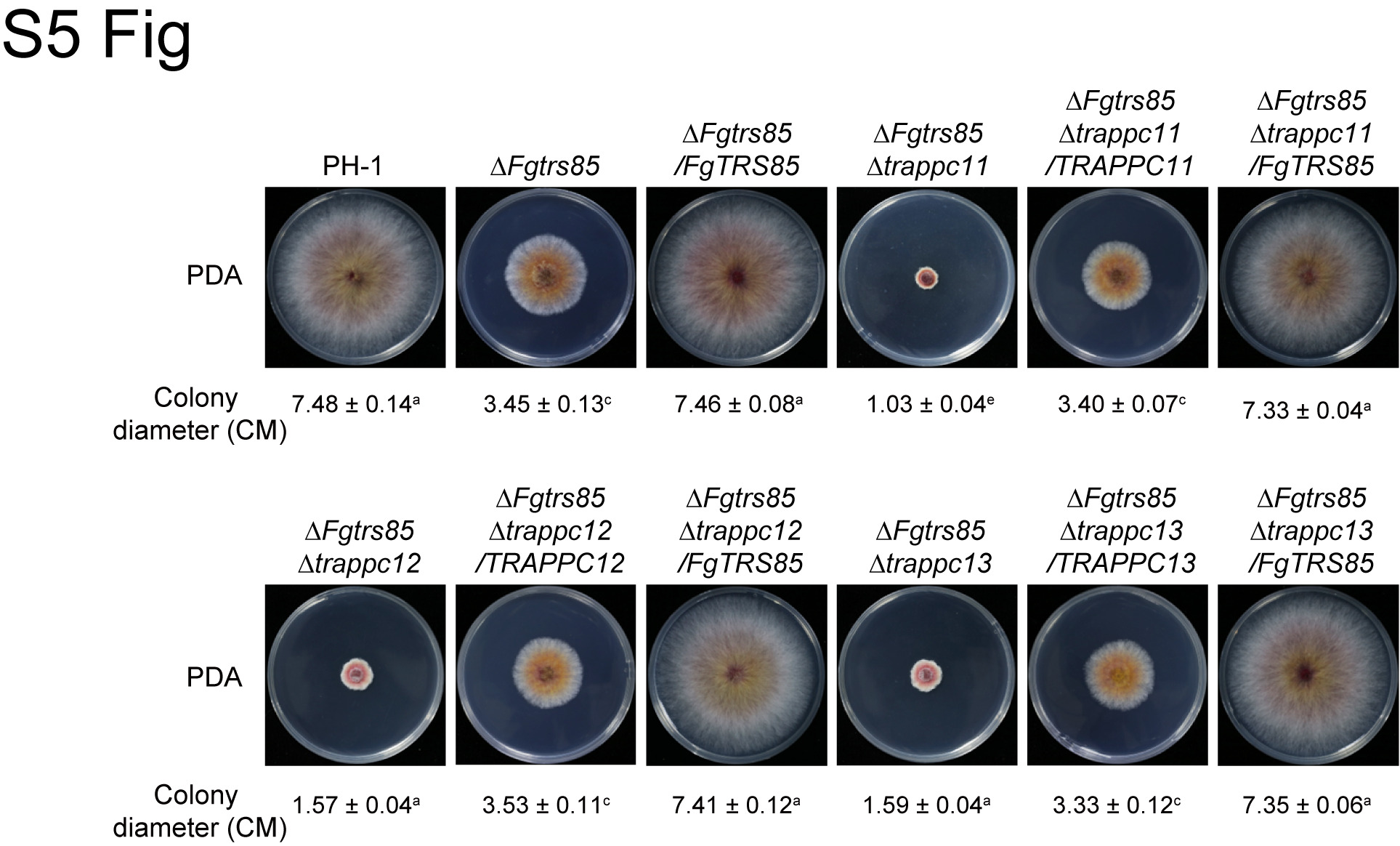

Supplement: S5 Fig — The colony growth and corresponding diameter measurements of the indicated strains were recorded after 3 days of culture on PDA. Data are from three independent experiments. ± represent SD. Different lowercase letters above the bars denote statistically significant differences (p < 0.01). (TIF) [file ppat.1013627.s005.tif]

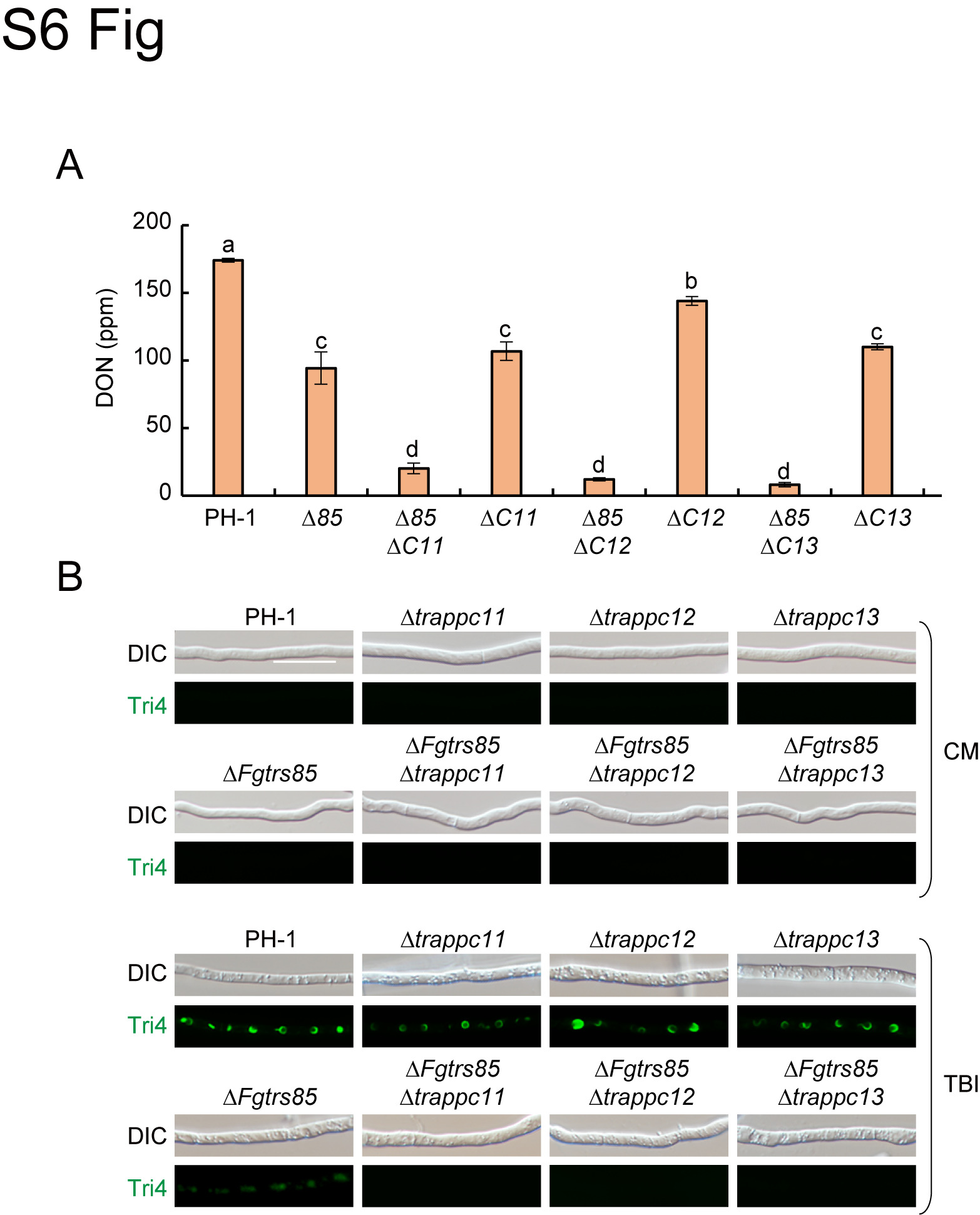

Supplement: S6 Fig — (A-B) Defective DON biosynthesis in TRAPPIII-specific subunits mutants. Levels of DON production were determined in wheat seeds infected with PH-1 and TRAPPIII-specific subunits mutant strains. ± represent SD. The same letters indicate no statistical significance, while different letters indicate significant difference (p < 0.01). The PH-1 and TRAPPIII-specific subunits mutant strains expressing Tri4-GFP were visualized by fluorescence microscopy under both CM and TBI medium. (TIF) [file ppat.1013627.s006.tif]

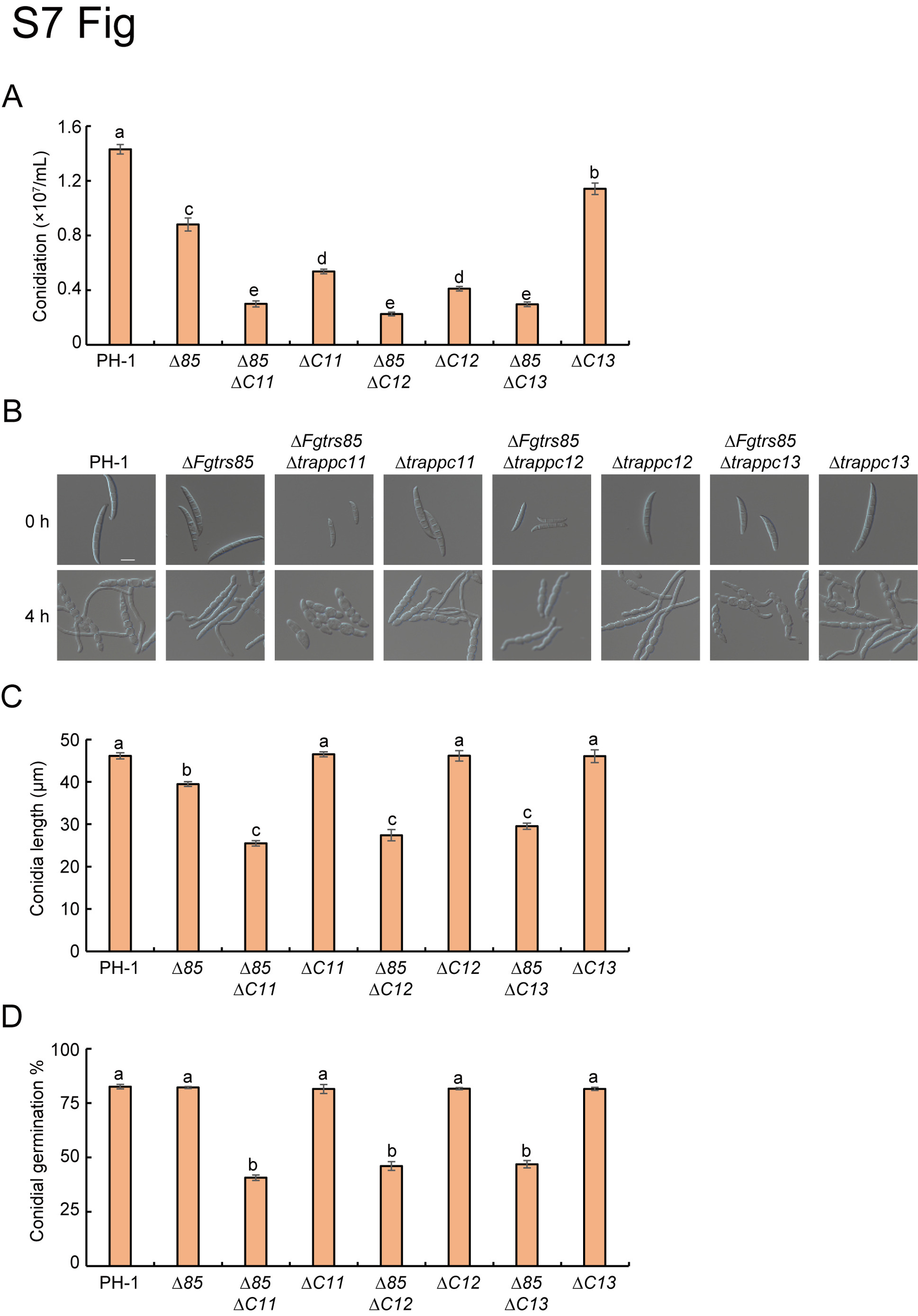

Supplement: S7 Fig — (A) TRAPPIII is involved in conidia production. PH-1 and mutant strains were cultured in CMC medium for 5 days to produce conidia. (B-D) TRAPPIII is involved in the germination of conidia. Conidia of PH-1 and mutant strains were incubated in liquid YEPD medium for 0 and 4 hours and conidial germination was detected by using live-cell microscopy. Conidial length and germination rates were quantified. ± represent SD. The same letters indicate no statistical significance, while different letters indicate significant difference (p < 0.01). (TIF) [file ppat.1013627.s007.tif]

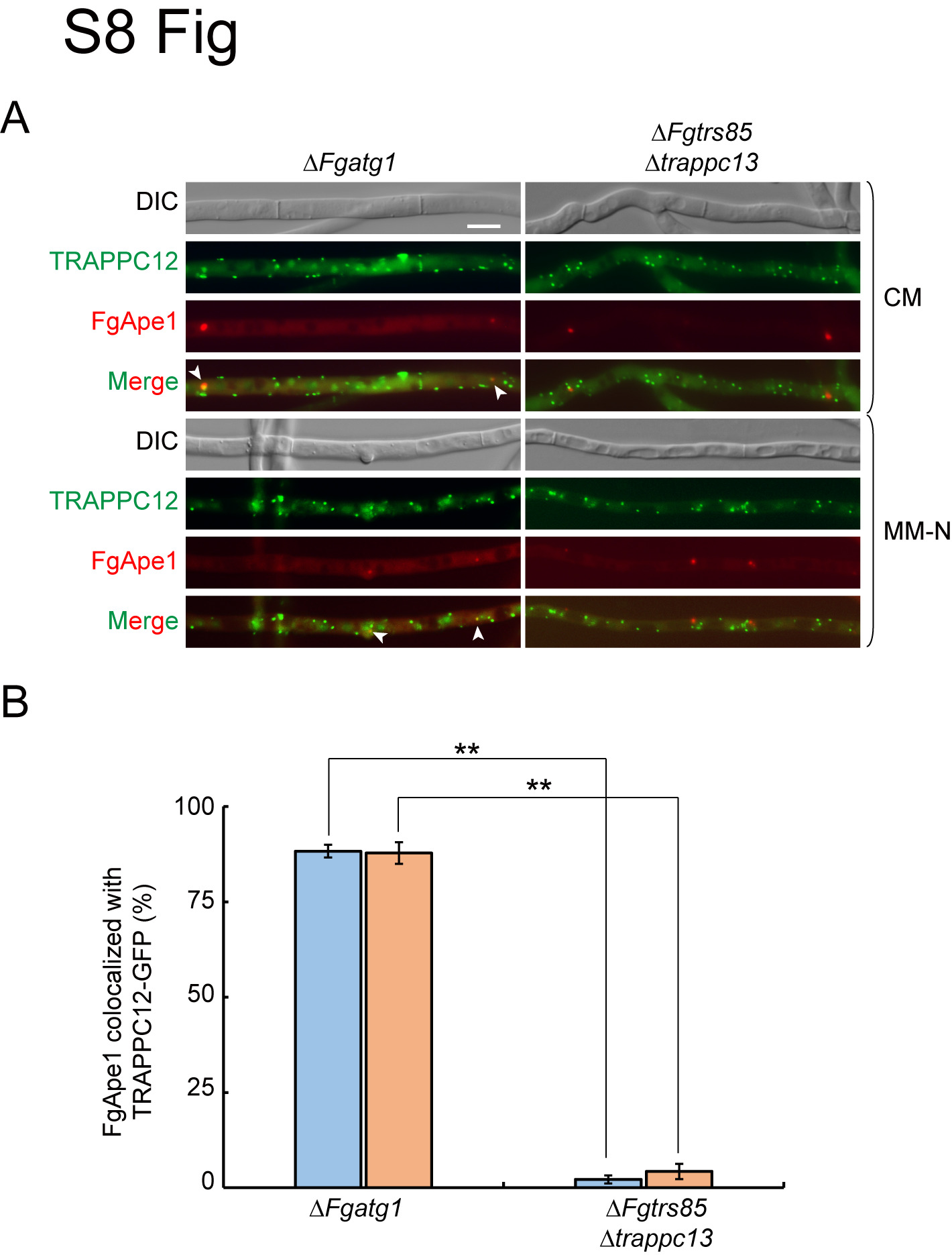

Supplement: S8 Fig — (A) The localization of TRAPPC12 to the PAS was examined in ΔFgatg1 and ΔFgatg1Δtrappc13 mutants. The strains co-expressing TRAPPC12-GFP and RFP-FgApe1 were examined by fluorescence microscopy under CM or MM-N conditions. Arrowheads indicate the PAS, marked by RFP-FgApe1 puncta, that colocalize with TRAPPC12-GFP. Bar = 10 μm. (B) The quantification of colocalization between RFP-FgApe1 and TRAPPC12-GFP from panel A is presented. More than 300 RFP-FgApe1 puncta were examined for each strain. Error bars represent SD. Results represent three independent experiments. **P < 0.01. (TIF) [file ppat.1013627.s008.tif]

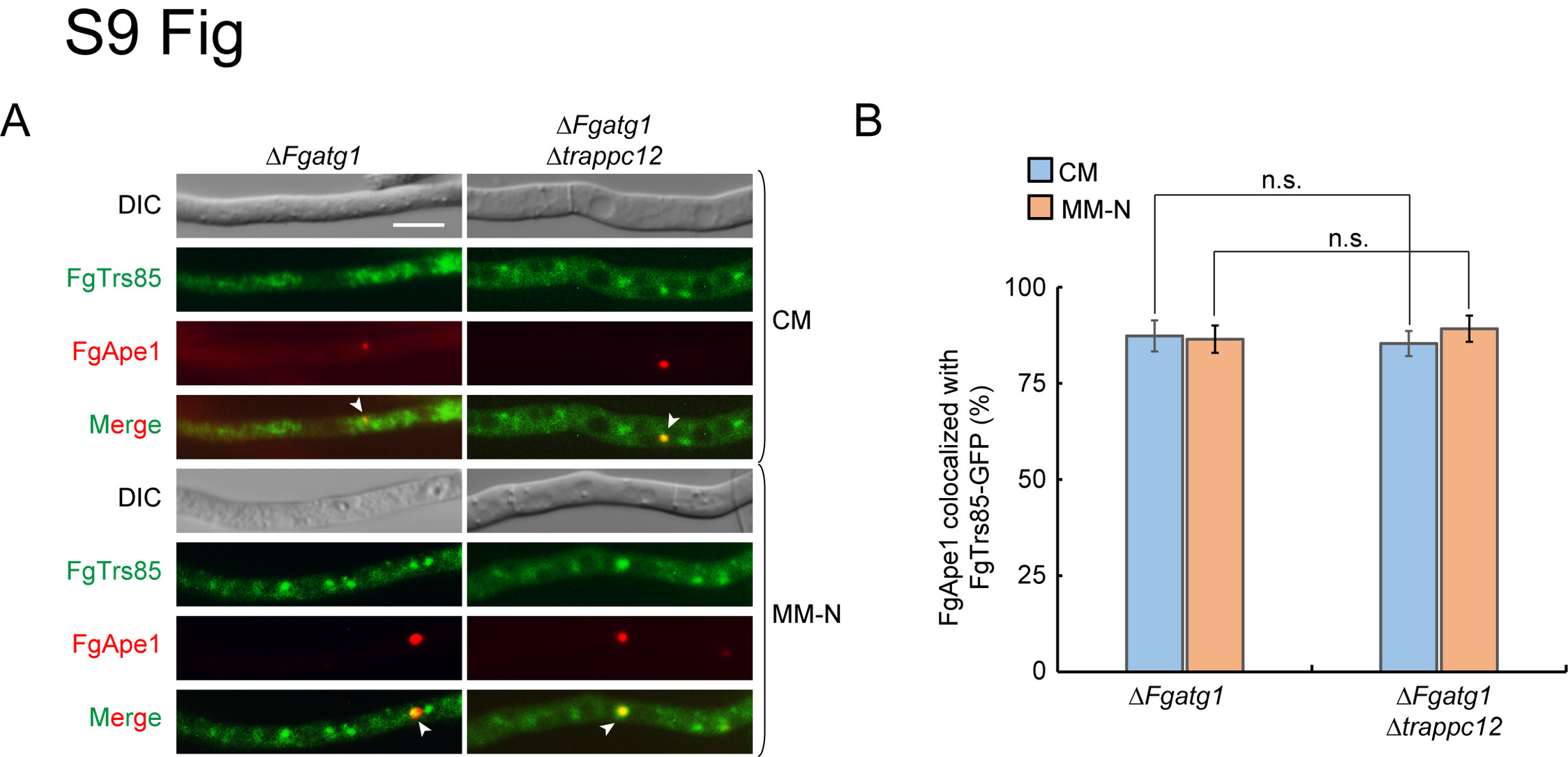

Supplement: S9 Fig — (A) The localization of FgTrs85 to the PAS in ΔFgatg1 and ΔFgatg1Δtrappc12 mutants was assessed. The indicated strains co-expressing FgTrs85-mNeoGreen and RFP-FgApe1 were examined by fluorescence microscopy under CM or MM-N conditions. Arrowheads indicate RFP-FgApe1 puncta that colocalize with FgTrs85-mNeoGreen. Bar = 10 μm. (B) Quantification of the colocalization between RFP-FgApe1 and FgTrs85-mNeoGreen from panel A. More than 300 RFP-FgApe1 puncta were examined for each strain. Error bars represent SD. Results represent three independent experiments. **P < 0.01. (TIF) [file ppat.1013627.s009.tif]

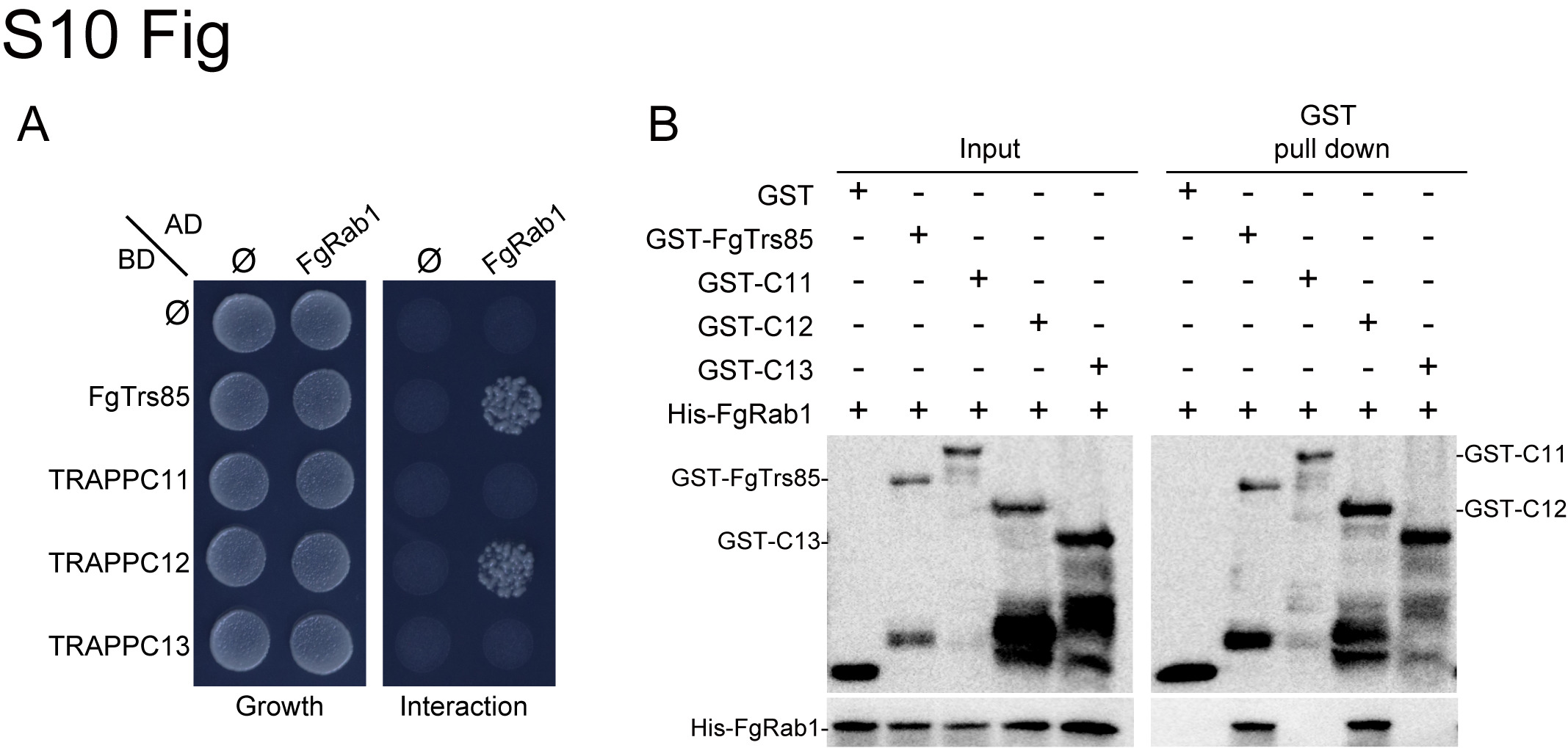

Supplement: S10 Fig — (A) FgRab1 interacts with FgTrs85 and TRAPPC12, but not with TRAPPC11 and TRAPPC13, in Y2H assay. Yeast cells were growth on agar plates of SD-Leu-Trp (growth) and SD-Leu-Trp-His-Ade (interaction). (B) A GST pull-down assay confirms the interaction between TRAPPIII-specific subunits and FgRab1 in vitro. GST-tagged FgTrs85, TRAPPC11, TRAPPC12 and TRAPPC13, along with His-tagged FgRab1, were expressed in E. coli. Western blot was performed using antibodies against GST and His. (TIF) [file ppat.1013627.s010.tif]

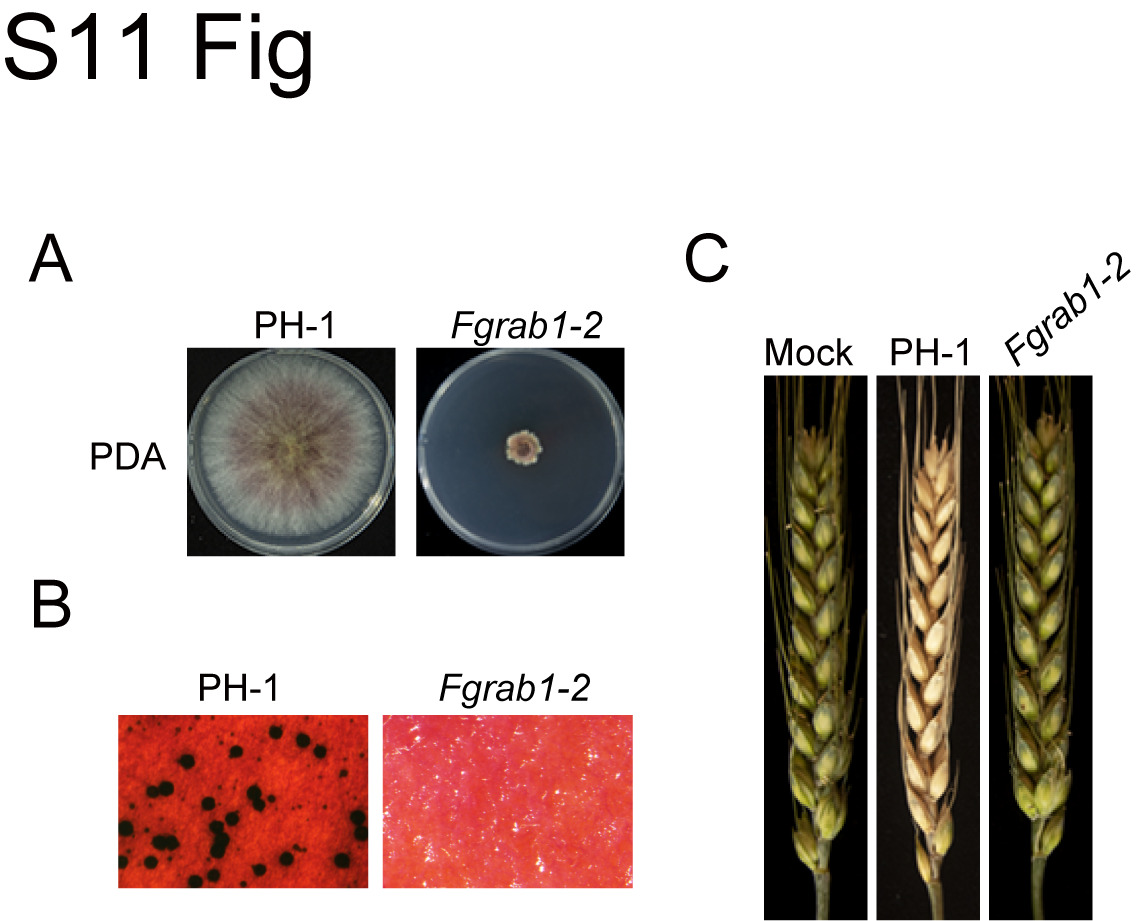

Supplement: S11 Fig — (A) The Fgrab1–2 mutant exhibit vegetative growth defects. PH-1 and Fgrab1–2 strains were cultured on PDA for 3 days. (B) The Fgrab1–2 mutant exhibit perithecia formation defects during the sexual reproduction. PH-1 and Fgrab1–2 mutant strains were cultured on carrot agar plates to detect perithecia production. (C) The Fgrab1–2 mutant exhibit virulence defects under plant infection. Conidial suspensions of the PH-1 and mutant strains were inoculated into flowering wheat heads. (TIF) [file ppat.1013627.s011.tif]

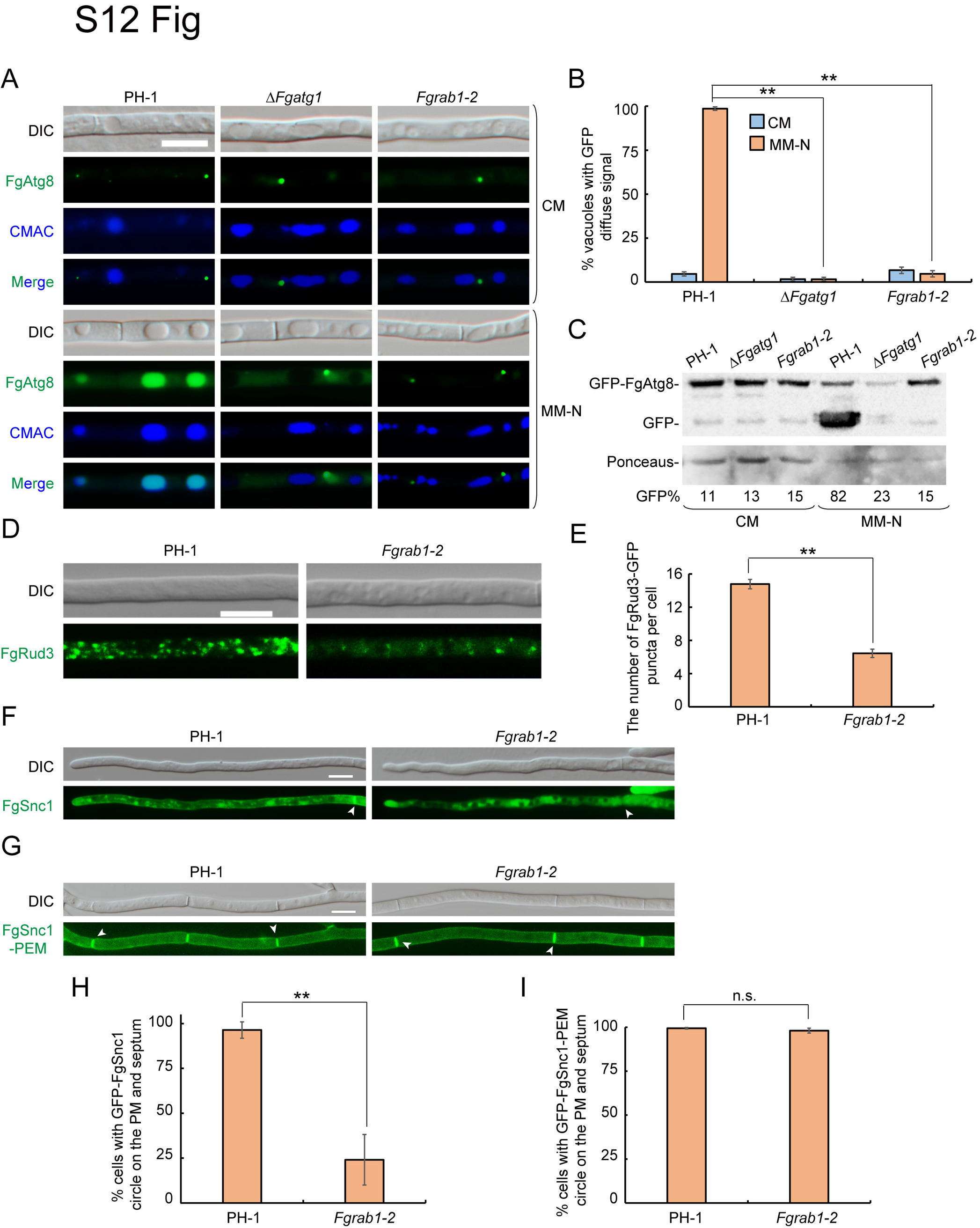

Supplement: S12 Fig — (A) The Fgrab1–2 mutant is defective in the transport of GFP-FgAtg8 to the vacuole. Hyphae of PH-1 and Fgrab1–2 expressing GFP-FgAtg8 were examined by fluorescence microscopy after growth in CM or MM-N medium. Vacuoles were stained with CMAC before visualization by microscopy. Bar = 10 μm. (B) Quantification of GFP-FgAtg8 transport to the vacuole from panel A. More than 200 cells were analyzed for each strain. Error bars represent SD. Results represent three independent experiments. **P < 0.01; N.S., no significance. (C) The Fgrab1–2 mutant exhibits a defect in GFP-FgAtg8 processing under starvation. Hyphae of WT and mutant strains expressing GFP-FgAtg8 were grown in CM medium and then shifted to MM-N. Protein extracts were resolved in vegetative hyphae lysates by immunoblot analysis using anti-GFP antibodies; the percentage of free GFP in each lane is shown under the blot. (D-E) FgRab1 is involved in the transport of FgRud3 to early Golgi. PH-1 and Fgrab1–2 strains expressing FgRud3-GFP were examined by fluorescence microscopy in CM medium. Bar = 10 μm. Quantification of the number of FgRud3-GFP dots in each strain from panel D, and more than 300 cells in each strain were examined. (F-I) FgRab1 is involved in the transport of FgSnc1 from endosome to late-Golgi. PH-1 and Fgrab1–2 strains expressing GFP-FgSnc1 or GFP-FgSnc1-PEM were examined by fluorescence microscopy in CM medium. Bar = 10 μm. The localization of GFP-FgSnc1 or GFP-FgSnc1-PEM with PM and septum were quantified from panel F or G, and more than 100 cells in each strain were examined. (TIF) [file ppat.1013627.s012.tif]

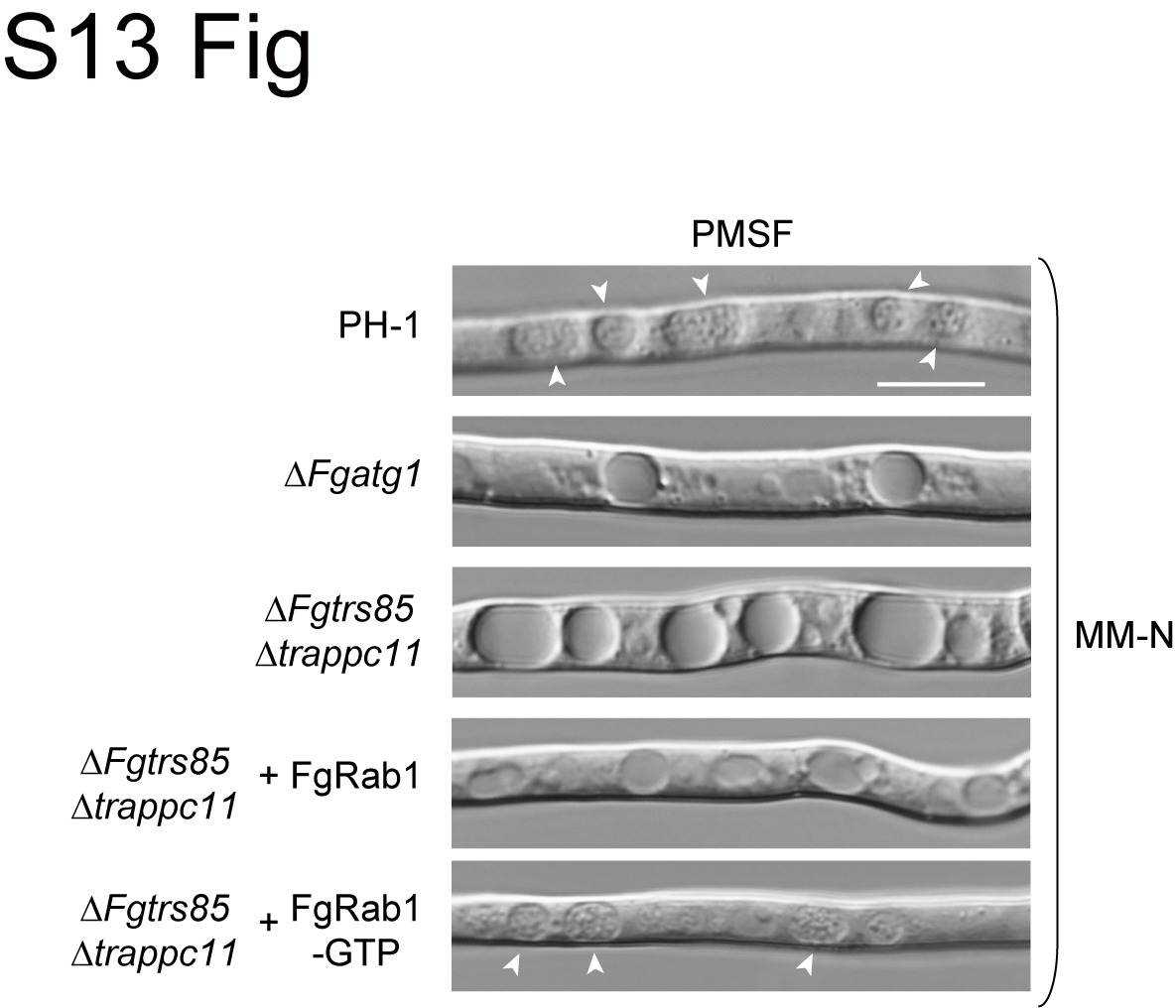

Supplement: S13 Fig — PH-1, ΔFgatg1, ΔFgtrs85ΔTRAPPC11, and ΔFgtrs85ΔTRAPPC11 strains overexpressing FgRab1 or FgRab1-GTP were grown in CM medium and then shifted to MM-N containing 2 mM of PMSF. Overexpression of FgRab1-GTP in ΔFgtrs85ΔTRAPPC11 and PH-1 hyphae accumulate autophagic bodies inside their vacuole. Arrowheads indicate autophagic bodies in the vacuole of hyphae. Bar = 10 μm. (TIF) [file ppat.1013627.s013.tif]
