# Supplementary material for: The TRAPPIII complex regulates development and virulence of Fusarium graminearum by coordinating autophagy and intracellular transport
Source: PLoS Pathog. 2025 Oct 24;21(10):e1013627. doi: 10.1371/journal.ppat.1013627 (PMC12578332; doi:10.1371/journal.ppat.1013627)
Supplement: S1 Table — (DOCX) [file ppat.1013627.s014.docx]

**S1 Table. *F. graminearum* strains used in this study**

| Strain | Genotype | | Source | | Figures | |
| --- | --- | --- | --- | --- | --- | --- |
| FDH69 | PH-1(Wide Type) | | (Wang et al. 2011)[1] | | Fig.2A-C Fig.8I Fig.9A-D Fig.S3B-C  Fig.S4B-D Fig.S5 Fig.S6A Fig.S7B  Fig.S11 Fig.S13 | |
| FDH378 | Δ*Fgtrs85#4* | | This study | | Fig.2A-C Fig.8I Fig.9A-D Fig.S5 Fig.S6A Fig.S7B | |
| FDH1849 | Δ*trappc11#3* | | This study | | Fig.2A-C Fig.8I Fig.S5 Fig.S6A Fig.S7B | |
| FDH1858 | Δ*trappc12#3* | | This study | | Fig.2A-C Fig.S5 Fig.S6A Fig.S7B | |
| FHD1870 | Δ*trappc13#5* | | This study | | Fig.2A-C Fig.S5 Fig.S6A Fig.S7B | |
| FHD2239 | Δ*Fgtrs85*Δ*trappc11#1* | | This study | | Fig.2A-C Fig.8I Fig.9A-B Fig.9D Fig.S5 Fig.S6A Fig.S7B Fig.S13 | |
| FHD2241 | Δ*Fgtrs85*Δ*trappc12#7* | | This study | | Fig.2A-C Fig.S5 Fig.S6A Fig.S7B | |
| FHD2242 | Δ*Fgtrs85*Δ*trappc13#1* | | This study | | Fig.2A-C Fig.S5 Fig.S6A Fig.S7B | |
| FHD2257 | Δ*trappc11*Δ*trappc12*Δ*trappc13#2* | | This study | | Fig.S4C-D | |
| FHD1930 | Δ*Fgatg1#1* | | This study | | Fig.2A Fig.2C Fig.S13 | |
| FHD2672 | *Fgrab1-2#3* | | This study | | Fig.S11 | |
| FHD3095 | PH-1/GFP-TRAPPC12+FgTrs85-tdTomato | | This study | | Fig.1C | |
| FHD147 | PH-1/GFP-FgAtg8 | | This study | | Fig.3A-B Fig.S12A Fig.S12C | |
| FHD461 | Δ*Fgatg1*/GFP-FgAtg8 | | This study | | Fig.3A-B Fig.5C Fig.S12A Fig.S12C | |
| FHD600 | Δ*Fgtrs85*/GFP-FgAtg8 | | This study | | Fig.3A-B | |
| FHD2352 | Δ*trappc11*/GFP-FgAtg8 | | This study | | Fig.3A-B | |
| FHD2353 | Δ*trappc12*/GFP-FgAtg8 | | This study | | Fig.3A-B | |
| FHD2354 | Δ*trappc13*/GFP-FgAtg8 | | This study | | Fig.3A-B | |
| FHD601 | Δ*Fgtrs85*Δ*trappc11*/GFP-FgAtg8 | | This study | | Fig.3A-B Fig.5C | |
| FHD594 | Δ*Fgtrs85*Δ*trappc12*/GFP-FgAtg8 | | This study | | Fig.3A-B | |
| FHD599 | Δ*Fgtrs85*Δ*trappc13*/GFP-FgAtg8 | | This study | | Fig.3A-B | |
| FHD3058 | Δ*Fgatg1*/GFP-TRAPPC12+mCherry-FgApe1 | | This study | | Fig.4A | |
| FHD3061 | Δ*Fgtrs85*/GFP-TRAPPC12+mCherry-FgApe1 | | This study | | Fig.4A | |
| FHD3064 | Δ*Fgtrs85*Δ*trappc11*/GFP-TRAPPC12+mCherry-FgApe1 | | This study | | Fig.4A | |
| FHD3082 | PH-1/GFP-Atg8+mCherry-FgApe1 | | This study | | Fig.5A | |
| FHD3085 | Δ*Fgatg1*/GFP-FgAtg8+mCherry-FgApe1 | | This study | | Fig.5A | |
| FHD3088 | Δ*Fgtrs85*/GFP-FgAtg8+mCherry-FgApe1 | | This study | | Fig.5A | |
| FHD3091 | Δ*Fgtrs85*Δ*trappc11*/GFP-FgAtg8+mCherry-FgApe1 | | This study | | Fig.5A | |
| FHD2754 | Δ*Fgrab7*/GFP-Atg8#2 | | This study | | Fig.5C | |
| FHD3074 | Δ*Fgatg1*/GFP-TRAPPC12+mCherry-FgAtg8 | | This study | | Fig.5D | |
| FHD3076 | Δ*Fgrab7*/GFP-TRAPPC12+mCherry-FgAtg8 | | This study | | Fig.5D | |
| FHD2261 | PH-1/FgAtg9-GFP+mCherry-FgApe1 | | This study | | Fig.6C | |
| FHD2263 | Δ*Fgatg1*/FgAtg9-GFP+mCherry-FgApe1 | | This study | | Fig.6C | |
| FHD2262 | Δ*Fgtrs85*/FgAtg9-GFP+mCherry-FgApe1 | | This study | | Fig.6C | |
| FHD2347 | Δ*Fgatg1*Δ*Fgtrs85*/FgAtg9-GFP+mCherry-FgApe1 | | This study | | Fig.6C | |
| FHD3078 | Δ*Fgtrs85*Δ*trappc11*/FgAtg9-GFP+mCherry-FgApe1 | | This study | | Fig.6C | |
| FHD2350 | PH-1/FgTrs85-GFP+mCherry-FgSed5 | | This study | | Fig.7A | |
| FHD2351 | PH-1/FgTrs85-GFP+FgKex2-mCherry | | This study | | Fig.7A | |
| FHD2348 | PH-1/FgTrs85-GFP+FgVps21-tdTomato | | This study | | Fig.7A | |
| FHD3111 | PH-1/GFP-TRAPPC12+mCherry-FgSed5 | | This study | | Fig.7C | |
| FHD3114 | PH-1/GFP-TRAPPC12+FgKex2-mCherry | | This study | | Fig.7C | |
| FHD3107 | PH-1/GFP-TRAPPC12+FgVps21-tdTomato | | This study | | Fig.7C | |
| FHD1726 | PH-1/FgSec22-GFP | | This study | | Fig.8C | |
| FHD3120 | Δ*Fgtrs85*/FgSec22-GFP | | This study | | Fig.8C | |
| FHD3117 | Δ*trappc11*/FgSec22-GFP | | This study | | Fig.8C | |
| FHD3123 | Δ*Fgtrs85*Δ*trappc11*/FgSec22-GFP | | This study | | Fig.8C | |
| FHD3034 | PH-1/FgRud3-GFP | | This study | | Fig.8A Fig.S12D | |
| FHD3040 | Δ*Fgtrs85*/FgRud3-GFP | | This study | | Fig.8A | |
| FHD3037 | Δ*trappc11*/FgRud3-GFP | | This study | | Fig.8A | |
| FHD3044 | Δ*Fgtrs85*Δ*trappc11*/FgRud3-GFP | | This study | | Fig.8A | |
| FHD2180 | PH-1/Rp27-GFP-FgSnc1 | | This study | | Fig.8E Fig.S12F | |
| FHD3141 | Δ*Fgtrs85*/Rp27-GFP-FgSnc1 | | This study | | Fig.8E | |
| FHD3138 | Δ*trappc11*/Rp27-GFP-FgSnc1 | | This study | | Fig.8E | |
| FHD3145 | Δ*Fgtrs85*Δ*trappc11*/Rp27-GFP-FgSnc1 | | This study | | Fig.8E | |
| FHD2187 | PH-1/GFP-FgSnc1-PEM | | This study | | Fig.8G Fig.S12G | |
| FHD3131 | Δ*trappc11*/GFP-FgSnc1-PEM | | This study | | Fig.8G | |
| FHD3135 | Δ*Fgtrs85*/GFP-FgSnc1-PEM | | This study | | Fig.8G | |
| FHD3127 | Δ*Fgtrs85*Δ*trappc11*/GFP-FgSnc1-PEM | | This study | | Fig.8G | |
| FHD2259 | Δ*Fgtrs85*/RP27-FgRab1 | | This study | | Fig.9A-C | |
| FHD3155 | Δ*Fgtrs85*Δ*trappc11*/RP27-FgRab1 | | This study | | Fig.9A-B Fig.9D Fig.S13 | |
| FHD2667 | Δ*Fgtrs85*/RP27-FgRab11 | | This study | | Fig.9A-C | |
| FHD2670 | Δ*Fgtrs85*Δ*trappc11*/RP27-FgRab11 | | This study | | Fig.9A-B Fig.9D | |
| FHD581 | PH-1/Tri4-GFP | | This study | | Fig.S6B | |
| FHD2425 | Δ*trappc11*/Tri4-GFP | | This study | | Fig.S6B | |
| FHD2426 | Δ*trappc12*/Tri4-GFP | | This study | | Fig.S6B | |
| FHD2427 | Δ*trappc13*/Tri4-GFP | | This study | | Fig.S6B | |
| FHD1938 | Δ*Fgtrs85*/Tri4-GFP | | This study | | Fig.S6B | |
| FHD2428 | Δ*Fgtrs85*Δ*trappc11*/Tri4-GFP | | This study | | Fig.S6B | |
| FHD2430 | Δ*Fgtrs85*Δ*trappc12*/Tri4-GFP | | This study | | Fig.S6B | |
| FHD2432 | Δ*Fgtrs85*Δ*trappc13*/Tri4-GFP | | This study | | Fig.S6B | |
| FHD3470 | *Fgrab1-2*/GFP-FgAtg8 | | This study | | Fig.S12A Fig.S12C | |
| FHD3472 | *Fgrab1-2*/FgRud3-GFP | | This study | | Fig.S12D | |
| FHD3483 | *Fgrab1-2*/Rp27-GFP-FgSnc1 | | This study | | Fig.S12F | |
| FHD3486 | *Fgrab1-2*/GFP-FgSnc1-PEM | | This study | | Fig.S12G | |
| FHD3788 | PH-1/HIS-C11+GFP-Trs85 | | This study | | Fig.S2 | |
| FHD3790 | PH-1/HIS-C12+GFP-Trs85 | | This study | | Fig.S2 | |
| FHD3792 | PH-1/HIS-C13+GFP-Trs85 | | This study | | Fig.S2 | |
| FHD3501 | Δ*Fgatg1*/C12-GFP+Ape1-mcherry | | This study | | Fig.S8A | |
| FHD3798 | Δ*Fgtrs85*Δ*TRAPPC13*/C12-GFP+mCherry-Ape1 | | This study | | Fig.S8A | |
| FHD3800 | Δ*Fgatg1*/mNeongreen-Trs85+mCherry-Ape1 | This study | | Fig.S9A | |  |
| FHD3796 | Δ*Fgatg1*Δ*trappc12*/mNeongreen-Trs85+mCherry-Ape1 | This study | | Fig.S9A | |  |

References

1. Wang C, Zhang S, Hou R, Zhao Z, Zheng Q, Xu Q, Zheng D, Wang G, Liu H, Gao X, Ma JW, Kistler HC, Kang Z, and Xu JR. 2011. Functional analysis of the kinome of the wheat scab fungus *Fusarium graminearum*. PLoS Pathog 7: e1002460.
