# Supplementary material for: The TRAPPIII complex regulates development and virulence of Fusarium graminearum by coordinating autophagy and intracellular transport
Source: PLoS Pathog. 2025 Oct 24;21(10):e1013627. doi: 10.1371/journal.ppat.1013627 (PMC12578332; doi:10.1371/journal.ppat.1013627)
Supplement: S2 Table — (DOCX) [file ppat.1013627.s015.docx]

**S2 Table. Primers used in this study**

| Primer name | Oligonucleotide sequence (5’-3’) | Remark |
| --- | --- | --- |

| AD-*FgTRS85*-F | GCTCATATGGCCATGGAGGCCGAATTCATGCCACAACCTCTCGAAAATG | Construction of FgTrs85 Y2H vector |
| --- | --- | --- |
| AD-*FgTRS85*-R | GGATCCCGTATCGCCCGGGAATTCCTATGTACCATCATCAGTGGTACTGCC |  |
| BD-*FgTRS85*-F | GGACCTGCATATGGCCATGGAGGCCGAATTCATGCCACAACCTCTCGAAAATG |  |
| BD-*FgTRS85*-R | GCTGCAGGTCGACGGATCCCCGGGAATTCCTATGTACCATCATCAGTGGTACTGCC |  |
| AD-*TRAPPC11*-F | CGCTCATATGGCCATGGAGGCCAGTGAATTCATGGATGGGTATCCGACGGG | Construction of TRAPPC11 Y2H vector |
| AD-*TRAPPC11*-R | CGATGGATCCCGTATCGATGCCCACCCGGGTGGAATTCTCACTGCGCCTCCGAC |  |
| BD-*TRAPPC11*-F | GGAGGACCTGCATATGGCCATGGAGGCCGAATTCATGGATGGGTATCCGACGGG |  |
| BD-*TRAPPC11*-R | GCGGCCGCTGCAGGTCGACGGATCCCCGGGAATTCTCACTGCGCCTCCGAC |  |
| AD-*TRAPPC12*-F | CATATGGCCATGGAGGCCAGTGAATTCATGAGTGAACCTAAAAGAACAGGAAGTC | Construction of TRAPPC12 Y2H vector |
| AD-*TRAPPC12*-R | GGATCCCGTATCGATGCCCACCCGGGTGGAATTCTCAACCCCTTTCGGTGCG |  |
| BD-*TRAPPC12*-F | GCATATGGCCATGGAGGCCGAATTCATGAGTGAACCTAAAAGAACAGGAAGTC |  |
| BD-*TRAPPC12*-R | GCGGCCGCTGCAGGTCGACGGATCCCCGGGAATTCTCAACCCCTTTCGGTGCG |  |
| AD-*TRAPPC13*-F | CGCTCATATGGCCATGGAGGCCAGTGAATTCATGAGCCATCAAAGATATCCCTCCC | Construction of TRAPPC13 Y2H vector |
| AD-*TRAPPC13*-R | GGATCCCGTATCGATGCCCACCCGGGTGGAATTCTCATCGTGCCGCTCGTC |  |
| BD-*TRAPPC13*-F | CCTGCATATGGCCATGGAGGCCGAATTCATGAGCCATCAAAGATATCCCTCCC |  |
| BD-*TRAPPC13*-R | GCGGCCGCTGCAGGTCGACGGATCCCCGGGAATTCTCATCGTGCCGCTCGTC |  |
| AD-*FgRAB1*-F | CGCTCATATGGCCATGGAGGCCAGTGAATTCTTGAGCCTCTCTCTCAACTCTCTG | Construction of FgRab1 Y2H vector |
| AD-*FgRAB1*-R | CGATGGATCCCGTATCGATGCCCACCCGGGTGGAATTCTGGGCTGGTTGTCAAGCTG |  |
| *FgTRS85*-A-F | CCGTTTCTTCTTCCTTGGCT | For amplify *FgTRS85*A fragment |
| *FgTRS85*-A-R | GACAGGGAACAGCAGCTCG |  |
| *FgTRS85*-B-F | GACCAATGACCAAAAGGCAG | For amplify *FgTRS85*B fragment |
| *FgTRS85*-B-R | CGCGATCGGCAGTGTAGTA |  |
| *TRAPPC11*-A-F | GGATATCCCGTGTGTCAAAGCAC | For amplify *TRAPPC11*A fragment |
| *TRAPPC11*-A-R | GATGTGTTGACCTCCACTAGCTCCAGCCAAGCCGGGCCAGCCCACTTACAAG |  |
| *TRAPPC11*-B-F | GGAATAGAGTAGATGCCGACCGCGGGTTGTATGACAAGACGTTGTGCCAATG | For amplify *TRAPPC11*B fragment |
| *TRAPPC11*-B-R | CCCTTCTCCCCTTTCCTCATC |  |
| *TRAPPC12*-A-F | GCCTTTCAGAGCCATGGC | For amplify *TRAPPC12*A fragment |
| *TRAPPC12*-A-R | GATGTGTTGACCTCCACTAGCTCCAGCCAAGCCCAGCATCGCAGGTTTTAAACATCG |  |
| *TRAPPC12*-B-F | GGAATAGAGTAGATGCCGACCGCGGGTTCCCAATACACGACCGTATGGTTTC | For amplify *TRAPPC12*B fragment |
| *TRAPPC12*-B-R | CAATGCAACGAGGAGCCC |  |
| *TRAPPC13*-A-F | GACCCTTACGCTGCCTATGG | For amplify *TRAPPC13*A fragment |
| *TRAPPC13*-A-R | GCATTGATGTGTTGACCTCCACTAGCTCCAGCCAAGCCGCCGACCTGGAAATGCGAC |  |
| *TRAPPC13*-B-F | GGGCAAAGGAATAGAGTAGATGCCGACCGCGGGTTGACATTCTGTCCACGATGCG | For amplify *TRAPPC13*B fragment |
| *TRAPPC13*-B-R | CGTGCGTTTCAGGTTACTGTCC |  |
| H1-F | GGCTTGGCTGGAGCTAGTGGAGGTCAA | For amplify hygromycin H1 fragment |
| H1-R | AACCCGCGGTCGGCATCTACTCTATTC |  |
| H2-F | GATGTAGGAGGGCGTGGATATGTCCT | For amplify hygromycin H2 fragment |
| H2-R | GTATTGACCGATTCCTTGCGGTCCGAA |  |
| *FgTRS85*-M-F | GCTTCGACGCTTTCACCG |  |
| *FgTRS85*-M-R | CATAGTGCGGCAGAACCG |  |
| *FgTRS85*-K1-F | AACTTCTCGCGTCGCTTG |  |
| *FgTRS85*-K2-R | ACTACTGGTGTCTTGGCAACTCT |  |
| *TRAPPC11*-M-F | CGGTGCCACCAACGAC | For identification of *TRAPPC11* deletion transformants |
| *TRAPPC11*-M-R | GCTTGCGGGCTCGAAAC |  |
| *TRAPPC11*-K1-F | GGAGAATGGGGTATCGAACGAG |  |
| *TRAPPC11*-K2-R | CCGGGAAGGAAAGGGAGAG |  |
| *TRAPPC12*-M-F | GCCCCTCAGCGTACTTCC | For identification of *TRAPPC12* deletion transformants |
| *TRAPPC12*-M-R | GGGTGTTCAAATGCTGCGC |  |
| *TRAPPC12*-K1-F | CAGTTTGGTTGCTCGCCG |  |
| *TRAPPC12*-K2-R | GCCCGCTCTCCTAAGAGG |  |
| *TRAPPC13*-M-F | GATTCCGGCTTCACTCGCG | For identification of *TRAPPC13* deletion transformants |
| *TRAPPC13*-M-R | CCGCAACCACAAAACAGACC |  |
| *TRAPPC13*-K1-F | GATCGTAACCATTCCCACGG |  |
| *TRAPPC13*-K2-R | GCTTCCCGTCTACCGGG |  |
| *TRAPPC11*-A-R(Nat) | CCAAAATAAGCATTGATGTGTTGACCTCCCCCGGGCCAGCCCACTTACAAG | For amplify A, B fragment of double knockout |
| *TRAPPC11*-B-F(Nat) | CTTAAATAAATACTACTCAGTAATAACGTATGACAAGACGTTGTGCCAATG |  |
| *TRAPPC12*-A-R(Nat) | CAAAATAAGCATTGATGTGTTGACCTCCCCCCAGCATCGCAGGTTTTAAACATCG |  |
| *TRAPPC12*-B-F(Nat) | CTTAAATAAATACTACTCAGTAATAACCCCAATACACGACCGTATGGTTTC |  |
| *TRAPPC13*-A-R(Nat) | CTAAACCAAAATAAGCATTGATGTGTTGACCTCCCCCGCCGACCTGGAAATGCGAC |  |
| *TRAPPC13*-B-F(Nat) | CAATACTTAAATAAATACTACTCAGTAATAACGACATTCTGTCCACGATGCGTG |  |
| N1-F | GGGGGAGGTCAACACATCAATG | For amplify nourseothricin N1 fragment |
| N1-R | GTCGTACAGGGCGGTGTCC |  |
| N2-F | CCTGACCAAGGTGTTCCCC | For amplify nourseothricin N2 fragment |
| N2-R | GTTATTACTGAGTAGTATTTATTTAAGTATTG |  |
| *TRAPPC12*-GFP-F | GATCACTCTCGGCATGGACGAGCTGTACAAGATGAGTGAACCTAAAAGAACAGGAAGT | For GFP-TRAPPPC12 fusion construct generation |
| *TRAPPC12*-GFP-R | GCCTGAATGTTGAGTGGAATGATGGGATCCAAGCTCGAGTCAACCCCTTTCGGTGCGG |  |
| GFP-*FgTRS85*-F | GGGATCACTCTCGGCATGGACGAGCTGTACAAGATGCCACAACCTCTCGAAAATGAAG | Construction of GFP-FgTrs85 vector |
| GFP-*FgTRS85*-R | GTTGAGTGGAATGATCTCGAGCTATGTACCATCATCAGTGGTACTGCC |  |
| GFP(85)-F | GATTTAAATCGTGGTTCTCATCACCATCACCATCACTCGAGGTGAGCAAGGGCGAGGAG |  |
| GFP(85)-R | GTCGGGGTAGCTTCATTTTCGAGAGGTTGTGGCATCTTGTACAGCTCGTCCATGCC |  |
| *FgTRS85*-mNeongreen-F | GATGTGATGGGCATGGACGAGCTGTACAAGATGCCACAACCTCTCGAAAATGAAG | Construction of FgTrs85-mNeongreen vector |
| *FgTRS85*-mNeongreen-R | GTTGAGTGGAATGATCTCGAGCTATGTACCATCATCAGTGGTACTG |  |
| mNeongreen-F | CGTGGTTCTCATCACCATCACCATCACTCGAGGTGAGCAAGGGCGAGGAG |  |
| mNeongreen-R | GGGGTAGCTTCATTTTCGAGAGGTTGTGGCATCTTGTACAGCTCGTCCATGCC |  |

| *TRI1*-QF | TTGAACACTACCTCGGTGCT | For *TRI* gene qRT-PCR analysis |
| --- | --- | --- |
| *TRI1*-QR | AGTTCGCGAGCATTCTTGAC |  |
| *TRI4*-QF | CCTGGTCTGGTCACCATTCT |  |
| *TRI4*-QR | ATGGCCAGTGTCCTTGAAGT |  |
| *TRI5*-QF | GAGTGTTTCATGCATGGCTACGTC |  |
| *TRI5*-QR | CTGAGCCTCCTTCACATCGTCC |  |
| *TRI6*-QF | CTGAGGGCATTCTGAGTAGCGACA |  |
| *TRI6*-QR | CGTTATGTTTATCGGCACTTTG |  |
| *TRI10*-QF | GCGACAGGAGCAAGAACATAA |  |
| *TRI10*-QR | GGCGGCGTAAATCTGAGTG |  |
| GAPDH-F | CTTACTGCCTCCACCAACTG |  |
| GAPDH-R | TGACGTTGGAAGGAGCGAAG |  |
| Pro-*FgRAB11*(DT)-F | CGTGGTTCTCATCACCATCACCATCACTCGAGCCTACAGCACCTACCCTAGGC | Construction of *Fgrab1-2* |
| Pro-*FgRAB11*(DT)-R | GAAGCGGAGAGTGGCGTACTACTCGGGGTTCATTGTGGGATGTGGAGAGGGAG |  |
| *FgRAB1*(DT)-F1 | GAGCCGCGCATTCTCTCCCTCTCCACATCCCACAATGAACCCCGAGTAGTACGC |  |
| *FgRAB1*(DT)-R2 | TGTTGAGTGGAATGATGGGATCCAAGCTCGAGTTAGCAGCAGCTGTTGTTGGAAG |  |
| *FgRAB1*(G83E)-R1 | GTTGAAAGAGTCCATGTCGGTAACGTCGTAGACGACGCAAATTTCGTGGGCGCCGC |  |
| *FgRAB1*(G83E)-F2 | CAATTACCTCTTCGTACTACCGCGGCGCCCACGAAATTTGCGTCGTCTACGACGTTAC |  |
| *TRAPPC11*-GST-F | gcgaccatcctccaaaatcggatggttcaactagtATGGATGGGTATCCGACGGG | Construction of pulldown vector |
| *TRAPPC11*-GST-R | ccaactcagcttcctttcgggctttgtttaaagcttCTGCGCCTCCGACTTCTCA |  |
| *TRAPPC12*-GST-F | ccaaaatcggatggttcaactagtATGAGTGAACCTAAAAGAACAGGAAGTCAAG |  |
| *TRAPPC12*-GST-R | cagccaactcagcttcctttcgggctttgtttaaagcttACCCCTTTCGGTGCGG |  |
| *TRAPPC13*-GST-F | atcctccaaaatcggatggttcaactagtATGAGCCATCAAAGATATCCCTCCCA |  |
| *TRAPPC13*-GST-R | ccaactcagcttcctttcgggctttgtttaaagcttTCGTGCCGCTCGTCTAGTC |  |
| *FgATG9*-GST-F | CCAAAATCGGATCTGGTTCCGCGTGGATCCATGGCATCAAACATATTCTCCCGGA |  |
| *FgATG9*-GST-R | GGCAGATCGTCAGTCAGTCACGATGCGGCCGCTCGAGTACCATTCCGCCGCCTCG |  |
| *FgTRS85*-HIS-F | gacaaggccatggctgatatcggatccgaattcATGCCACAACCTCTCGAAAATGAAG |  |
| *FgTRS85*-HIS-R | ggtggtggtggtgctcgagtgcggccgcaagcttTGTACCATCATCAGTGGTACTGCC |  |
| *TRAPPC11*-HIS-F | cgacgacaaggccatggctgatatcggatccgaattcATGGATGGGTATCCGACGGG |  |
| *TRAPPC11*-HIS-R | ggtggtggtggtggtgctcgagtgcggccgcaagcttTCACTGCGCCTCCGACTTC |  |
| *TRAPPC12*-HIS-F | ggccatggctgatatcggatccgaattcATGAGTGAACCTAAAAGAACAGGAAGTC |  |
| *TRAPPC12*-HIS-R | ggtggtggtggtggtgctcgagtgcggccgcaagcttACCCCTTTCGGTGCGG |  |
| *TRAPPC13*-HIS-F | ggccatggctgatatcggatccgaattcATGAGCCATCAAAGATATCCCTCCC |  |
| *TRAPPC13*-HIS-R | ggtggtggtggtggtgctcgagtgcggccgcaagcttTCGTGCCGCTCGTCTAGT |  |
| HIS-*FgRAB1*-F | CAAGGCCATGGCTGATATCGGATCCGAATTCATGAACCCCGAATACGACTATCTCTTC |  |
| HIS-*FgRAB1*-R | GTGGTGGTGGTGGTGCTCGAGTGCGGCCGCAAGCTTTTAGCAGCAGCTGTTGTTGGAAG |  |
| GST-*TRS85*-F | ggcgaccatcctccaaaatcggatggttcaactagtATGCCACAACCTCTCGAAAATG |  |
| GST-*TRS85*-R | ctcagcttcctttcgggctttgtttaaagcttTGTACCATCATCAGTGGTACTGCC |  |
| *FgTRS85*-tdTomato-F | CGTGGTTCTCATCACCATCACCATCACTCGAGAGCAGACGAAACCGGGTTATAAATC | Construction of FgTrs85-tdTomato vector |
| *FgTRS85*-tdTomato-R | CTCTTTGATGACCTCCTCGCCCTTGCTCACCATTGTACCATCATCAGTGGTACTGCC |  |
| *FgATG8*-pro-F | GGTTCTCATCACCATCACCATCACTCGAGACTGAGAACTCGGGTGATAGTCAG | Construction of pro-GFP-FgAtg8 vector |
| *FgATG8*-pro-R | CGGTGAACAGCTCCTCGCCCTTGCTCACCATGTTGACGGTGATGGTTGTTGTG |  |
| GFP-F | CTTACCGCCTCCACAACAACCATCACCGTCAACATGGTGAGCAAGGGCGAG |  |
| GFP-R | GGGGTGCTCGTCCTTGAATTTGCTGCGCATCTTGTACAGCTCGTCCATGCC |  |
| *FgATG8*-F | GATCACTCTCGGCATGGACGAGCTGTACAAGATGCGCAGCAAATTCAAGGAC |  |
| *FgATG8*-R | GAGTGGAATGATGGGATCCAAGCTCGAGTTACGCTTCGCCAAAAGTGTTCTC |  |
| *FgAPE1*-mCherry-F | TCCCCCGTCCGCCAGCTCGCGTGCAGCTCTATCACACACAATGTTGAGCAAGGGCGAGG | Construction of RFP-FgApe1 vector |
| *FgAPE1*-mCherry-R | GCACTCAACATTGCAGGCGTAACTTGAGTCATAGCAGCCTTGTACAGCTCGTCCATGCC |  |
| *FgATG9*-GFP-F | GGTTCTCATCACCATCACCATCACTCGAGATGGCATCAAACATATTCTCCCGG | Construction of FgAtg9-GFP vector |
| *FgATG9*-GFP-R | CCCCGGTGAACAGCTCCTCGCCCTTGCTCACCATTACCATTCCGCCGCCTCG |  |
| HIS-*TRAPPC11*-F | TCACCATCACCATCACTCGAGCACCACCACCACCACCACATGGATGGGTATCCGACGGG | Construction of His-TRAPPC11 vector |
| HIS-*TRAPPC11*-R | GTGGAATGATCTCGAGTCAGTGGTGGTGGTGGTGGTGTCCCCTCTGTCGGTATTATGCG |  |
| HIS-*TRAPPC1*2-F | ATCACTCGAGCACCACCACCACCACCACATGAGTGAACCTAAAAGAACAGGAAGTCAAG | Construction of His-TRAPPC12 vector |
| HIS-*TRAPPC1*2-R | AATGATCTCGAGTCAGTGGTGGTGGTGGTGGTGTACGCGTTCCATAGTGAATACTTCCG |  |
| HIS-*TRAPPC1*3-F | ATCACCATCACTCGAGCACCACCACCACCACCACATGAGCCATCAAAGATATCCCTCCC | Construction of His-TRAPPC13 vector |
| HIS-*TRAPPC1*3-R | GAGTGGAATGATCTCGAGCTAGTGGTGGTGGTGGTGGTGGTCCCCAGCTTTCCGGTAGC |  |
