# Supplementary material for: The TRAPPIII complex regulates development and virulence of Fusarium graminearum by coordinating autophagy and intracellular transport
Source: PLoS Pathog. 2025 Oct 24;21(10):e1013627. doi: 10.1371/journal.ppat.1013627 (PMC12578332; doi:10.1371/journal.ppat.1013627)
Supplement: S3 Table — (DOCX) [file ppat.1013627.s016.docx]

**S3 Table. A list of putative FgTrs85-interacting proteins under starvation-induced condition identified by mass spectrometry assay**

| Proteins | Protein Description | Virulence |
| --- | --- | --- |
| FGSG_08748 | hypothetical protein | Not documented |
| FGSG_04046 | hypothetical protein | Not documented |
| FGSG_12900 | hypothetical protein | Not documented |
| FGSG_13660 | hypothetical protein | Reduced virulence |
| FGSG_09891 | arsenical pump-driving ATPase | Not documented |
| FGSG_02649 | trafficking protein particle complex subunit 3 | Not documented |
| FGSG_10873 | GTP-binding protein ypt1 | Reduced virulence |
| FGSG_06920 | ADP-ribosylation factor | Reduced virulence |
| FGSG_04393 | hypothetical protein | Reduced virulence |
| FGSG_06941 | hypothetical protein | Not documented |
| FGSG_02505 | trafficking protein particle complex subunit 6B | Not documented |
| FGSG_10138 | endoplasmic reticulum vesicle protein 25 precursor | Not documented |
| FGSG_01398 | hypothetical protein | Reduced virulence |
| FGSG_01044 | hypothetical protein | Not documented |
| FGSG_12896 | hypothetical protein | Not documented |
| FGSG_05164 | inositolphosphorylceramide-B C-26 | Not documented |
| FGSG_09850 | hypothetical protein | Not documented |
| FGSG_01890 | hypothetical protein | Not documented |
| FGSG_12029 | hypothetical protein | Not documented |
| FGSG_05141 | GTP-binding protein ypt7 | Reduced virulence |
